# CH_4_ activation by PtX^+^ (X = F, Cl, Br, I)

**DOI:** 10.3389/fchem.2022.1027465

**Published:** 2022-09-26

**Authors:** Jin Zhao, Lingxi Qi, Wenzuo Li, Jianbo Cheng, Qingzhong Li, Shaoli Liu

**Affiliations:** College of Chemisty and Chemical Engineering, Yantai University, Yantai, China

**Keywords:** PtX +, activation of methane, reaction mechanism, ligand effect, noncovalent interactions

## Abstract

Reactions of PtX^+^ (X = F, Cl, Br, I) with methane have been investigated at the density functional theory (DFT) level. These reactions take place more easily along the low-spin potential energy surface. For HX (X = F, Cl, Br, I) elimination, the formal oxidation state of the metal ion appears to be conserved, and the importance of this reaction channel decreases in going as the sequence: X = F, Cl, Br, I. A reversed trend is observed in the loss of H_2_ for X = F, Cl, Br, while it is not favorable for PtI^+^ in the loss of either HI or H_2_. For HX eliminations, the transfer form of H is from proton to atom, last to hydride, and the mechanisms are from PCET to HAT, last to HT for the sequence of X = F, Cl, Br, I. One reason is mainly due to the electronegativity of halogens. Otherwise, the mechanisms of HX eliminations also can be explained by the analysis of Frontier Molecular Orbitals. While for the loss of H_2_, the transfer of H is in the form of hydride for all the X ligands. Noncovalent interactions analysis also can be explained the reaction mechanisms.

## 1 Introduction

Catalysts that can convert methane directly into higher-value-added commodities have long been sought, but breaking the thermodynamically strong, kinetically inert C-H bonds in a controlled way under mild conditions remains a central challenge (Geng et al., 2017). Reactivity studies of transition-metal ions in the gas phase, and, in particular, aspects related to the ongoing challenge of selective activation of inert C-H and C-C bonds, have been studied intensely over the past decades (Howell and Burkinshaw, 1983; Dubois, 1989; Eller and Schwarz, 1991; Balcells et al., 2010; Dobereine and Crabtree, 2010; Roithova and Schröder, 2010; Jana et al., 2011). In recent years, how ligation affects the electronic structure at the transition-metal center has been systematically investigated (Howell and Burkinshaw, 1983; Dubois, 1989; Schlangen et al., 2007; Schlangen et al., 2007; Schlangen and Schwarz, 2008; Dede et al., 2009; Li et al., 2016a; Sun et al., 2016; Zhou et al., 2016; Zhou et al., 2017a; Zhou et al., 2017b; Zhou et al., 2017c; Schwarz et al., 2017; Schwarz et al., 2017; Yue et al., 2017). The ligand can change the electronic structure of the metal center through a shift in the electronic state, or provide a more efficient reaction center, so the addition of a single ligand to a metal center has been widely used to prepare reactants for C-H bonds activation (Chen et al., 1997; Rodgers et al., 2000; Li et al., 2009).

Irikura and Beauchamp (Irikura and Beauchamp, 1989; Irikura and Beauchamp, 1991a; Irikura and Beauchamp, 1991b) discovered that Pt^+^ as a 5d transition metal dehydrogenates methane to yield the corresponding carbene complexes Pt (CH_2_)^+^. Bare Pt^+^ also has been found to catalyze the reaction of methane with molecular oxygen in the gas phase to produce methanol, formaldehyde and other oxidation products (Wesendrup et al., 1994). Subsequently, a series of activation studies around transition metal Pt^+^ were carried out (Achatz et al., 2000; Wheeler et al., 2016). Recently, it has been reported that Pt^−^ is able to selectively activate one C-H bond in methane, which represents the first example of methane activation by atomic anions (Liu et al., 2019).

Open-shell ligands X form a covalent bond with the metal cation and thereby increase the formal oxidation state, for example, X = F, Cl, Br, I, OH, NH, O (Schlangen et al., 2007; Dede et al., 2009), which often increases reactivity. For example, bare Cr^+^ is one of the least reactive transition metal cations, whereas CrCl^+^ is significantly more reactive (Mandich et al., 1986). Clearly, this example demonstrates that an appropriately chosen ligand can enhance the selectivity of a reagent at the expense of reactivity (Schlangen et al., 2007). Similarly, the naked cations M^+^ (M = Fe, Co, Ni, Ru, Rh, Pd) do not bring about thermal C-H bond activation of methane (Halle et al., 1982; Tolbert and Beauchamp, 1986; Tolbert et al., 1986; Schultz et al., 1988; Musaev et al., 1993; Musaev and Morokuma, 1994; Westerberg and Blomberg, 1998), but the corresponding MH^+^ cations (Schilling et al., 1986; Elkind and Armentrout, 1987; Schilling et al., 1987; Schilling et al., 1987; Ohanessian et al., 1990; Zhang and Bowers, 2004; Li et al., 2009; Wang and Andrews, 2009) give rise to efficient H/CH_3_ ligand switches.

It is not surprising that the nature of the ligand X controls the outcome of a given ion-molecule reaction, as, for example, demonstrated in a systematic investigation of FeX^+^ cations with acetone (Schröder et al., 1993). The number of ligands also affects the reaction activity. With respect to the activation of methane, CrF^+^ is not sufficient, and CrF_2_^+^ does not react with CH_4_, whereas CrF_3_^+^ and CrF_4_^+^ are able to activate the C-H bonds of methane (Mazurek et al., 1998).

Schlangen *et al.* have reported the studies on ligand and substrate effects in gas-phase reactions of NiX^+^/RH couples (X = F, Cl, Br, I; R = CH_3_, C_2_H_5_, n-C_3_H_7_, n-C_4_H_9_) (Schlangen et al., 2007). The results indicate that NiF^+^ is the only Ni^Ⅱ^ halide complex that brings about thermal activation of methane to eliminate HF, whereas the nickel-halide cations NiCl^+^, NiBr^+^, and NiI^+^ react only with large alkanes. In the elimination of HX (X = F, Cl, Br, I), the formal oxidation state of the metal ion appears to be conserved, and the importance of this reaction channel decreased in going from NiF^+^ to NiI^+^. A reversed trend is observed in the losses of H_2_, which dominate the gas-phase ion chemistry of NiI^+^/RH couples. Schröder and Schwarz (2005) reported the reactions of methane with PtX^+^ (X = H, Cl, Br and CHO) using mass spectrometry and found that these species are able to activate methane.

Here, we report our calculated results for the PtX^+^/CH_4_ (X = F, Cl, Br, I) systems. The key issues for our study are the mechanistic details of methane catalyzed by ligated transition metal PtX^+^/CH_4_ (X = F, Cl, Br, I).

## 2 Computational and technical details

Full optimization of geometries for all stationary points involved in methane dehydrogenation by PtX^+^ (X = F, Cl, Br, I) has been calculated using the density functional theory (DFT) method based on the hybrid of Becke’s three-parameter exchange functional and the Lee, Yang, and Parr correlation functional (B3LYP) (Becke, 1988; Lee et al., 1988; Becke, 1993), Becke hybrid with correlation functional Perdew (B3P86) (Perdew, 1986a; Perdew, 1986b; Michael et al., 2008) and M06-2X (Zhao and Truhlar, 2008; Zhao and Truhlar, 2008). For carbon and hydrogen, also for F, Cl, and Br, the large 6-311+G** basis set is applied. The Stuttgart/Dresden relativistic effective core potentials (ECP) of SDD were adopted to describe the metal Pt and the halogen I (Andrae et al., 1990). For each optimized stationary point, vibrational analysis was performed at the same level with the geometry optimizations to determine its character (minimum or saddle point). Unscaled harmonic frequencies were employed to obtain entropy corrections and the zero-point vibrational energy (ZPVE) which is included in all relative energies. Furthermore, intrinsic reaction coordinate (IRC) calculations (Gonzalez and Schlegel, 1989) were performed to confirm that the optimized transition states correctly connect the relevant reactants and products. Energies were corrected for (unscaled) zero-point vibrational energy contributions and were given relative to the separated reactant couples PtX^+^/CH_4_ in the most stable spin state of PtX^+^ (Ye and Neese, 2010; Lawson Daku et al., 2012; Vargas et al., 2013). Between two different potential energy surfaces (PES), a configuration that structures are similar with almost the same energy was found, which is called minimum energy crossing point (MECP) (Poli and Harvey, 2003; Harvey, 2007). All computations reported are carried out using the GAUSSIAN 09 program suit (Frisch et al., 2009). The topological parameters of electron density(ρ), its Laplacian (▽^2^ρ), and energy density at the bond critical point (BCP) were analyzed with the AIM2000 program (Bader, 2000). The molecular electrostatic potentials (MEP) of the various monomers were calculated on the 0.001 a. u. isodensity surfaces using the wave function analysis–surface analysis suite (WFA-SAS) program (Bulat et al., 2010).

The geometries were optimized using density functional theory with B3P86, B3LYP, and M06-2X functional. The comparisons show that the results obtained by the three methods are very similar in terms of geometric optimization, energy, and potential energy surfaces. The data are shown in Table 1 and the Supporting Information. Among them, B3LYP shows a more systematic process in a high-spin state and is also more resource-efficient. Otherwise, we also calculated the single point energy of the reaction at the CCSD(T)/aug-cc-pVTZ (PP) level. The trend of the single point energy is similar to the previous potential energy surface except for the energy of ^1^Pt (CH_3_)^+^ in the last step, which is inconsistent with the experimental results (Schröder and Schwarz, 2005). So, all the data used are obtained based on the B3LYP method.

**TABLE 1:** Calculated relative energies (kcal/mol) of stationary points on the potential energy surfaces of the reaction PtF^+^ and CH_4_ in the singlet and triplet states in three methods.

	Singlet			Triplet		
	B3P86	B3LYP	M06-2X	B3P86	B3LYP	M06-2X
PtF^+^	0.00	7.48	32.14	1.22	0.00	0.00
PtF(CH_4_)^+^				−50.97	−36.92	−48.70
TS1				−37.08	−22.90	
PtHF(CH_3_)^+^	−62.23	−48.54	−57.00	−36.98	−23.01	−31.09
TS2	−42.49	−28.17	−39.15		−10.02	−13.73
Pt (CH_3_)(HF)^+^	−75.64	−63.91	−89.29		−60.40	−81.57
Pt (CH_3_)^+^+HF	−58.27	−47.61	−71.10		−45.93	−40.14
TS2-H_2_	−58.17	−42.28	−46.81	−12.36	2.83	−0.25
PtH_2_F(CH_2_)^+^	−58.00	−42.43	−46.39	−13.00	1.81	−0.56
TS3-H_2_	−54.65	−39.28	−42.92	−11.46	3.24	1.13
PtF(CH_2_)(H_2_)^+^	−54.72	−40.16	−44.52	−14.75	−3.78	−7.71
PtF(CH_2_)^+^+H_2_	−22.32	−12.79	−18.97	−5.80	2.89	−2.22

## 3 Results and discussions

**FIGURE 1:**
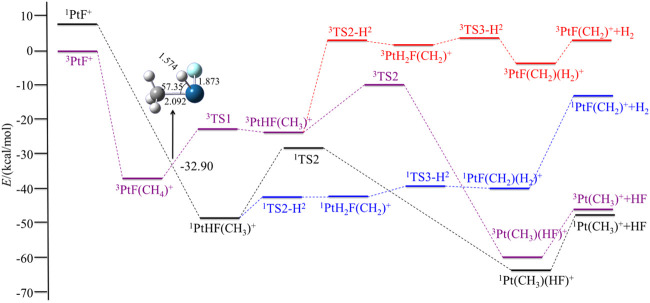
Potential energy surfaces of the reaction PtF^+^ + CH_4_ in the low- and high-spin states. The structure is the minimum energy crossing point (MECP).

In this section, we discuss the reactivity of the PtX^+^ (X = F, Cl, Br, I) in the activation process of CH_4_ and present a brief discussion of the most abundant or interesting processes for the PtX^+^/CH_4_ systems. Both low- and high-spin states have been considered. The potential energy surfaces of the reaction PtX^+^ + CH_4_ in the low- and high-spin states are summarized in Figure 1 and Figure 4, and the energetics (in kcal/mol) of the intermediates and transition states, relative to the ground state PtX^+^ plus CH_4_ have been summarized in Table 2. Geometries of these structures, including bond distances and bond angles, are summarized in the Supporting Information.

**TABLE 2:** Calculated relative energies (kcal/mol) of stationary points on the potential energy surfaces of the reaction PtX^+^ (X = F, Cl, Br, I) and CH_4_ in the singlet and triplet states.

Species	X = F		X = Cl		X = Br		X = I	
	Singlet	Triplet	Singlet	Triplet	Singlet	Triplet	Singlet	Triplet
PtX^+^	7.48	0.00	16.40	0.00	23.90	0.00	22.34	0.00
PtX (CH_4_)^+^		−36.92		−30.57		−26.39		−23.15
TS1		−22.90		−18.07		−13.60		−11.62
PtHX (CH_3_)^+^	−48.54	−23.01	−41.73	−18.43	−35.59	−14.02	−30.93	−12.21
TS2	−28.17	−10.02	−29.62	−2.10	−23.70	0.57	−15.54	6.21
Pt (CH_3_)(HX)^+^	−63.91	−60.40	−41.05	−27.47	−31.71	−15.00	−22.80	−4.92
Pt (CH_3_)^+^+HX	−47.61	−45.93	−10.45	−8.77	7.12	8.81	21.18	22.86
TS2-H_2_	−42.28	2.83	−26.52	8.85	−17.20	13.15	−8.94	12.57
PtH_2_X (CH_2_)^+^	−42.43	1.81	−26.61	8.57	−15.00	12.76	−9.11	11.28
TS3-H_2_	−39.28	3.24	−23.51	11.88	−14.42	16.47	−6.04	16.25
PtX (CH_2_)(H_2_)^+^	−40.16	−3.78	−24.84	7.28	−15.84	10.29	−7.56	11.15
PtX (CH_2_)^+^+H_2_	−12.79	2.89	−6.69	10.86	−0.74	14.78	3.97	16.85

The reactions observed in Figure 1 can be classified into two different categories: 1) reactions involving Pt-X bond cleavage, namely, the expulsions of HX and 2) bond activation of CH_4_ without obvious occurrence of Pt-X bond cleavage, that is, the loss of H_2_ (summarized in Eq. 1 and 2).

$${PtX}^{+}+{CH}_{4}\to Pt{\left({CH}_{3}\right)}^{+}+HX,$$ (1)

$${PtX}^{+}+{CH}_{4}\to PtX{\left({CH}_{2}\right)}^{+}+H_{2},$$ (2)

For the sake of simplicity, each species is labeled with its spin multiplicity as a superscript preceding the formula.

### 3.1 PtF^+^ + CH_4_

For PtF^+^, the ground electronic state has been found to be a triplet, and the singlet electronic excitation state of ^1^PtF^+^ has a relative energy of 7.48 kcal/mol. The reaction starts with the formation of a methane complex PtF(CH_4_)^+^. Based on Figure 1, the spin-conserving dehydrogenation of CH_4_ along the ground state route ^3^PtF^+^ + CH_4_ → ^3^PtF(CH_2_)^+^ + H_2_ is endothermic by 2.89 kcal/mol and cannot occur for the high efficiency of the reaction. So, the reaction would obtain HF through the ground route or would be a two-state reaction scenario (TSR) (Roithova et al., 2010).

In the triplet state, the relative energy of the complex ^3^PtF(CH_4_)^+^ is −36.92 kcal/mol and is found to have a η^2^ coordination, the θ_C-Pt-F_ = 179.89°, which indicates that the carbon atom attacks to Pt along the Pt-F axis. Then, Pt inserts into one of the C-H bonds of methane, resulting in a hydridomethyl complex ^3^PtHF(CH_3_)^+^, which has an energy of −23.01 kcal/mol. TS1 is the transition state of the oxidative addition of the first C-H bond on the reaction path. On the ^3^TS1, the activated C-H bond is almost broken with the C-H bond length of 1.775 Å and the Pt-H bond is nearly formed with the Pt-H bond length of 1.565 Å, indicating that ^3^TS1 is a typical three-centered late transition state, which is 14.02 kcal/mol above the encounter complex ^3^PtF(CH_4_)^+^ but only 0.11 kcal/mol above the ^3^PtHF(CH_3_)^+^.

The low-spin ^1^PtF^+^ with methane tends to form ^1^PtHF(CH_3_)^+^ intermediate directly. No activation transition state has been found on the singlet surface. The results indicate that the first C-H bond is activated spontaneously on the singlet surface. Energetically, the ^1^PtHF(CH_3_)^+^ is 25.53 kcal/mol lower than that of the triplet ^3^PtHF(CH_3_)^+^. A curve crossing is required from the triplet state to the singlet state *via* an MECP. As shown in Figure 1, due to the higher energies of the triplet-spin state in the process of the expulsions of HF, and the processes in the triplet-spin state being similar to the singlet-spin paths, later, the triplet surface is not considered in the expulsion of HF.

In the following pages of this section, we will first discuss the process of the expulsions of HX, namely, HF. On the singlet surface, the next step is a reductive elimination step to form an HF molecule complex; that is, the H and F rearrange to form an HF molecule electrostatically bound to Pt to obtain the ^1^Pt (CH_3_)(HF)^+^ with a barrier of 20.37 kcal/mol. In the last step, HF can be eliminated in an exothermic reaction by 17.98 kcal/mol. This last step leaves ^1^Pt (CH_3_)^+^ in its ground singlet state.

For the elimination of H_2_, a migration of hydrogen from CH_3_ to Pt, leading to ^1^PtH_2_F(CH_2_)^+^ with an energy barrier of 6.26 kcal/mol. The transition state ^1^TS2-H^2^ (it represents the transition state in the process of the elimination of H_2_) is a three-centered late transition state. Then, the two hydrogens rearrange easily to form the ^1^PtF(CH_2_)(H_2_)^+^. Afterward, the molecule H_2_ is eliminated. The calculated dissociation energy of H_2_ to ^1^PtF(CH_2_)^+^ is 27.38 kcal/mol.

Generally, the energy barrier controls the reaction rate in a channel. Comparing the above two reaction channels, the energy barrier of H_2_ elimination is 14.11 kcal/mol lower than that of the HF expulsions. However, in the subsequent steps, the calculated ligand dissociation energy of H_2_ to ^1^PtF(CH_2_)^+^ is much higher than any energies of the complexes in the path to produce HF. Namely, the favorable path for the reaction of PtF^+^ + CH_4_ is the channel of the elimination of HF.

For the overall process, the energetically most favorable route involves a two-state reactivity scenario. The favorable route is the elimination of HF *via* the route ^3^PtF^+^ + CH_4_ → ^3^PtF(CH_4_)^+^ → MECP → ^1^PtHF(CH_3_)^+^ → ^1^TS2 → ^1^Pt (CH_3_)(HF)^+^ → ^1^Pt (CH_3_)^+^+ HF.

### 3.2 PtX^+^ (X = Cl, Br, I) + CH_4_

As to the CH_4_ activation on PtX^+^ (X = Cl, Br, I), the mechanisms are very similar to those on PtF^+^, as discussed earlier. Indeed, the critical geometrical parameters in the intermediates and transition state are all very similar to the corresponding structures in the case of PtF^+^, as can be seen clearly by comparing the figures in the Supporting Information. Therefore, we shall not discuss their geometries in further detail but show some differences and their characteristics.

**FIGURE 2:**
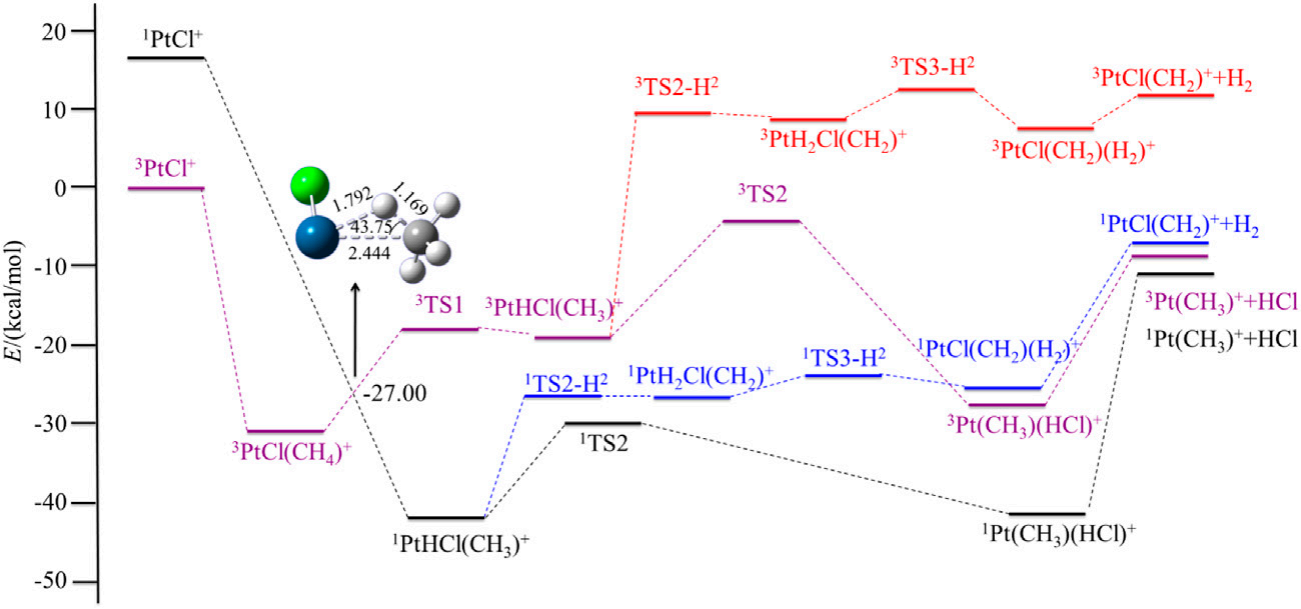
Potential energy surfaces of the reaction PtCl^+^ + CH_4_ in the low- and high-spin states. The structure is the minimum energy crossing point (MECP).

**FIGURE 3:**
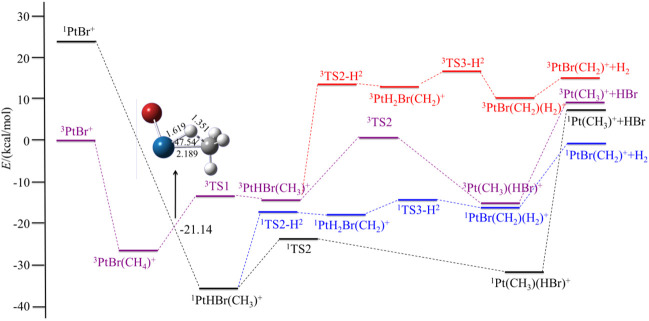
Potential energy surfaces of the reaction PtBr^+^ + CH_4_ in the low- and high-spin states. The structure is the minimum energy crossing point (MECP).

**FIGURE 4:**
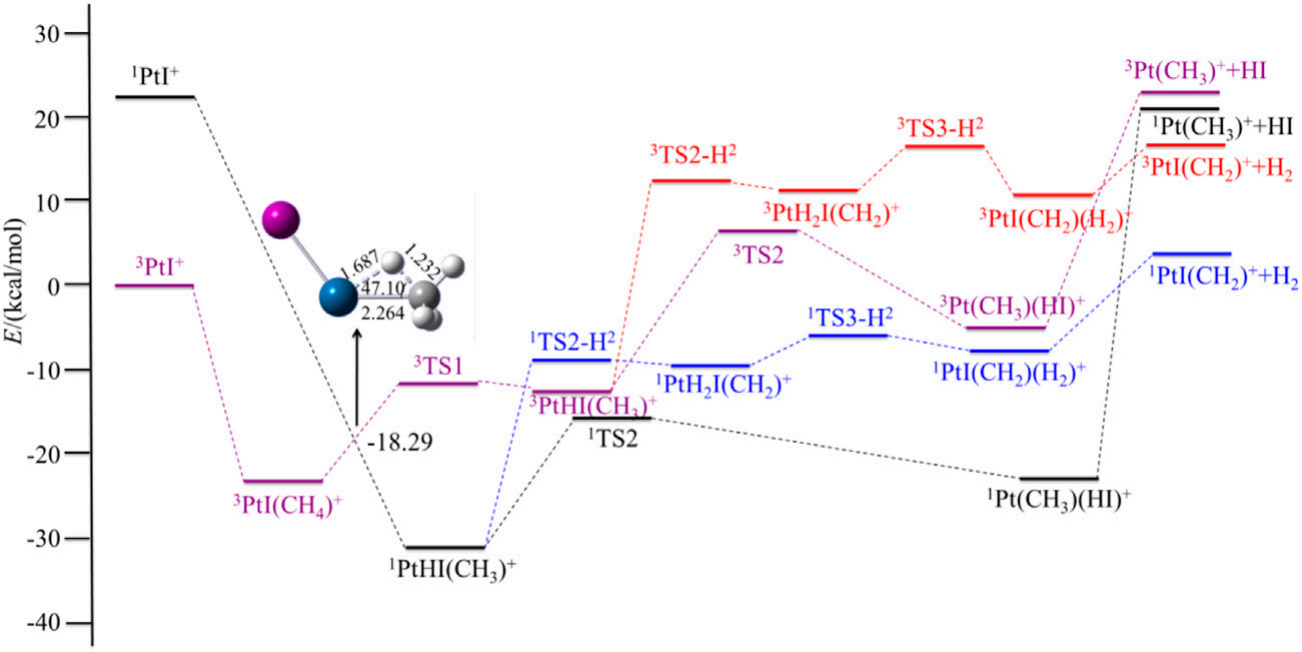
Potential energy surfaces of the reaction PtI^+^ + CH_4_ in the low- and high-spin states. The structure is the minimum energy crossing point (MECP).

For PtX^+^ (X = Cl, Br, I) with CH_4_, as calculated by the results, the ground low-lying state is all the triplet state. The excitation energies to the excited singlet state are 16.40, 23.90, and 22.34 kcal/mol, respectively, for PtX^+^ (X = Cl, Br, I). The low-spin ^1^PtX^+^ with methane tends to form a ^1^PtHX (CH_3_)^+^ intermediate directly. No activation transition state has been found on the singlet surface. The results are similar to the PtF^+^-CH_4_ system and indicate that C-H is activated spontaneously on the singlet surface. Energetically, the ^1^PtHX (CH_3_)^+^ is lower than the triplet ^3^PtHX (CH_3_)^+^. Since the triplet state is the ground state of PtX^+^, the methane activation starting from the ground state again requires an intersystem crossing as described in the case of PtF^+^ via a minimum energy crossing point, as shown in Figures 2–4.

Different from PtF^+^, as for the other three PtX^+^ (X = Cl, Br, I), on the singlet surface, the next step is the rearrangement of hydrogen and halogen to obtain HX, which has an activation energy of 12.11, 11.89, and 15.39 kcal/mol, respectively, for PtX^+^ (X = Cl, Br, I). This activation energy is 3.10, 6.50, and 6.60 kcal/mol lower than that in the path of the elimination of H_2_.

For PtCl^+^, HCl can be eliminated in an exothermic reaction by 10.45 kcal/mol. Due to the path of the expulsions of H_2_ always being high-lying compared with the process of elimination of HCl, it is an unfavorable path. Namely, the favorable path of PtCl^+^/CH_4_ is the process of HCl elimination. This result is in good agreement with the experimental results as reported by Schröder and Schwarz (2005). They reported the branching ratio of HCl is 100%. For PtBr^+^/CH_4_, the favorable path is the process of the elimination of HBr, but the calculated ligand dissociation energy of HBr to ^1^Pt (CH_3_)^+^ is 38.83 kcal/mol, different from PtF^+^, the energy difference between the two processes of the products are only 7.86 kcal/mol, which is lower than others. The activation energy to obtain ^1^Pt (CH_3_)(HBr)^+^ is 6.50 kcal/mol lower than to obtain ^1^PtH_2_Br(CH_2_)^+^. Due to the lower activation energy, the favorable path of the reaction of PtBr^+^/CH_4_ probably is the elimination of HBr, but also has some ratio of H_2_ in the products, as mentioned in the earlier discussions. As reported by Schröder and Schwarz (2005), the branching ratio of HBr:H_2_ is 85:15. As for PtI^+^, although the energies in the potential energy surfaces before the loss of HI are lower than that of H_2_, the elimination of HI needs much more energy than H_2_, and it is endothermic at 43.98 kcal/mol, which is not favorable to occur. In the process of the loss of H_2_, there is a barrier height of 21.99 kcal/mol on the singlet state to form the intermediate ^1^PtH_2_I(CH_2_)^+^. After overcoming a small barrier of 3.07 kcal/mol, the complex ^1^PtI(CH_2_)(H_2_)^+^ is formed. The low-spin species ^1^PtI(CH_2_)^+^ has an association energy of 11.53 kcal/mol. This reaction is endothermic of 3.97 kcal/mol, which is also not favorable thermodynamically.

In summary, in the elimination of HX (X = F, Cl, Br, I), the formal oxidation state of the metal ion appears to be conserved, and the importance of this reaction channel decreases in going as the sequence: X = F, Cl, Br, I. A reversed trend is observed in the loss of small closed-shell molecule H_2_ for X = F, Cl, Br, while it is not favorable for PtI^+^ in the loss of either HI or H_2_. The reason for the reactivity along with the abovementioned trends can be explained by the electronegative character of X; on the other hand, by the corresponding reaction enthalpies, which are mostly related to the formation of HX, that is to say, the halogens are heavier the bond-dissociation energies are much lower (Dede et al., 2009). The results can also be seen by the Natural Bond Orbital (NBO) populations and Natural population analysis (NPA) charge, as shown in the Supporting Information, and the results of part of the key structures are shown in Table 3. The electronegativity of the halogens decreases gradually from F to I, and the donor properties increase gradually from F to I, so F forms a strongly polarized covalent bond to Pt, and F increases the formal oxidation of Pt.

**TABLE 3:** Valence NBO populations for the 6s/5d/6p orbitals of Pt and the natural population analysis (NPA) charge of the related atoms in part of the key structures in the reaction of PtX^+^ (X = F, Cl, Br, I) + CH_4_ in the singlet state.

	PtF^+^				PtCl^+^				PtBr^+^				PtI^+^			
NBO																
TS2	0.42/8.73/0.01				0.43/8.93/0.01				0.41/9.01/0.01				0.42/9.07/0.02			
Pt (CH_3_)(HX)^+^	0.15/9.01/0.01				0.36/9.03/0.01				0.41/9.05/0.01				0.44/9.09/0.02			
TS2-H_2_	0.67/8.52/0.03				0.72/8.69/0.02				0.73/8.72/0.03				0.76/8.77/0.03			
PtX (CH_2_)(H_2_)^+^	0.57/8.57/0.02				0.62/8.71/0.02				0.64/8.74/0.01				0.67/8.82/0.02			
**NPA Charge**																
	**Pt**	**F**	**C**		**Pt**	**Cl**	**C**		**Pt**	**Br**	**C**		**Pt**	**I**	**C**	
TS2	0.85	−0.40	−0.52		0.64	−0.02	−0.52		0.57	0.12	−0.54		0.50	0.32	-0.55	
Pt (CH_3_)(HX)^+^	0.84	−0.54	−0.54		0.60	−0.02	−0.55		0.53	0.13	−0.56		0.46	0.34	-0.56	
TS2-H_2_	0.79	−0.52	−0.13		0.56	−0.25	−0.15		0.51	−0.15	−0.17		0.42	−0.01	-0.19	
PtX (CH_2_)(H_2_)^+^	0.84	−0.55	0.07		0.64	−0.26	0.02		0.59	-0.16	-0.004		0.48	−0.01	-0.06	
	**Pt**	**F**	**C**	**H**	**Pt**	**F**	**C**	**H**	**Pt**	**F**	**C**	**H**	**Pt**	**F**	**C**	**H**
PtHX (CH_3_)^+^	0.99	−0.38	−0.52	0.21	0.68	−0.08	−0.52	0.21	0.53	0.13	−0.56	0.20	0.50	0.17	−0.54	0.19
TS2	0.85	−0.40	−0.52	0.38	0.64	−0.02	−0.52	0.22	0.57	0.12	−0.54	0.19	0.50	0.32	−0.55	0.10
PtH_2_X (CH_2_)^+^	0.80	−0.55	−0.10	0.26	0.58	−0.28	−0.12	0.26	0.53	−0.19	−0.14	0.25	0.45	−0.05	−0.18	0.24
TS3-H_2_	0.80	−0.54	0.02	0.14	0.58	−0.27	−0.02	0.14	0.53	−0.18	−0.05	0.14	0.46	−0.06	−0.08	0.13

### 3.3 Mechanism discussions

Reaction mechanisms of these reactions are elucidated by detailed NPA charge and the Frontier Molecular Orbitals (HOMO and LUMO) of the key structures in the rate controlling step.

In the elimination of HF, as shown in Table 3, the F atom carries a significant negative charge, serving as a good proton acceptor, in which the electron is accepted by the metal center, thus, the NPA charge decreases in Pt. This process can be classified as a conventional proton-coupled electron transfer (PCET (Li et al., 2016b)) mechanism. For HCl and HBr eliminations, the charges of all atoms did not change during hydrogen transforms, and the mechanism can be judged as hydrogen atom transfer (HAT (Dietl et al., 2012)), while for I ligand, the NPA charge of I atom increases and that of H atom decreases, and it can be determined that the process is hydride transfer (HT (Li et al., 2016c)) mechanism.

**FIGURE 5:**
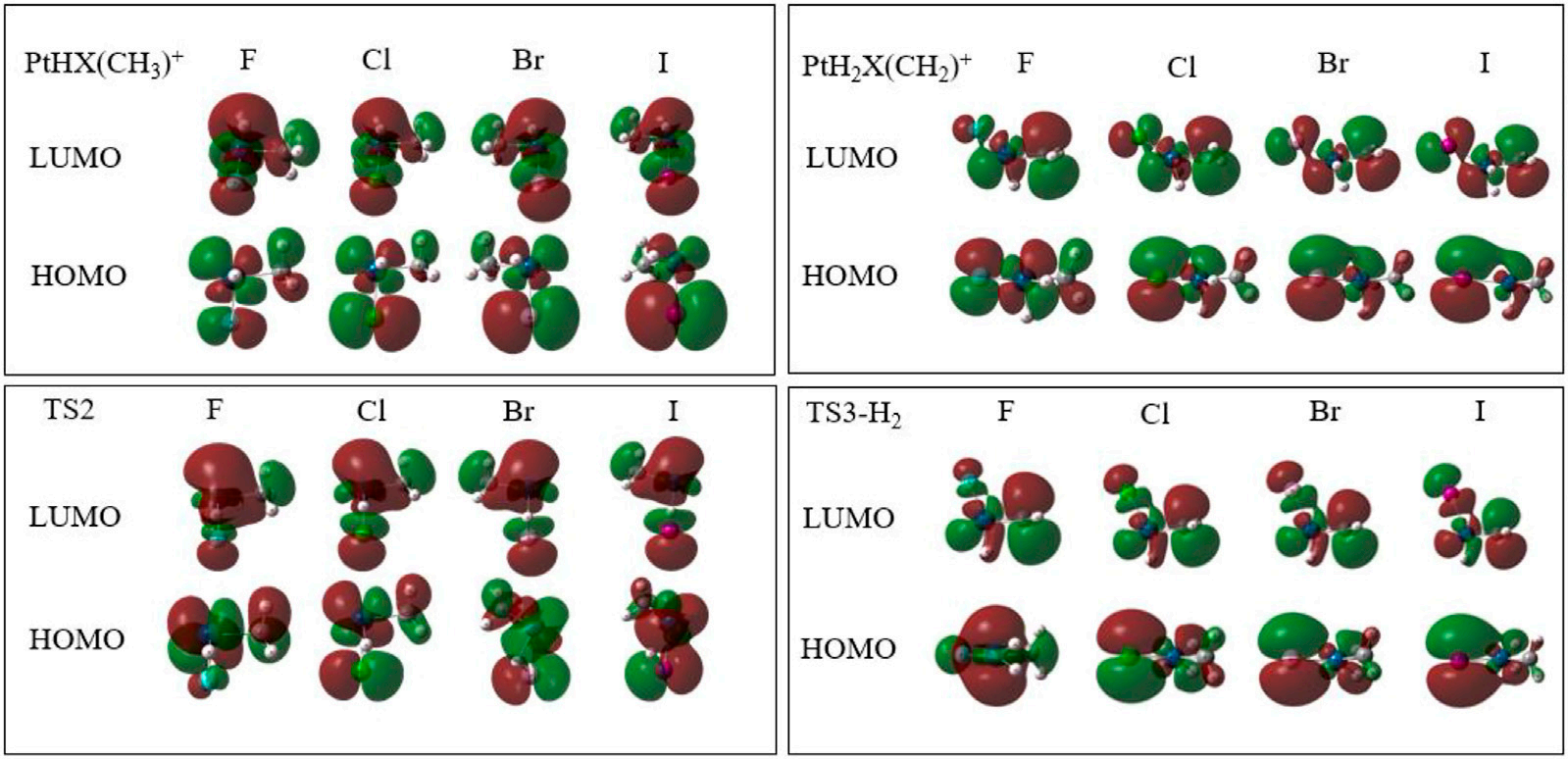
HOMO/LUMO orbital graphs in part of the structures of rate controlling step in the reaction of PtX^+^ (X = F, Cl, Br, I) + CH_4_ in the singlet state.

The mechanisms of HX eliminations also can be explained by the analysis of Frontier Molecular Orbitals, which are shown in Figure 5. In the HOMO orbital, the σ(d_x2-y2_) of Pt and σ(p_y_) of X occupied the main contribution in Pt-X molecular orbital. The coefficient of Pt (σ(d_x2-y2_)) becomes smaller and X (σ(p_y_)) increases in the sequence of F < Cl < Br < I. From ^1^PtHX (CH_3_)^+^ to ^1^TS2, the increase of electron density (φ^2^) on Pt is consistent with the decrease of NPA charge. The decrease of electron density on X also corresponds to the increase of NPA charge. It also can be seen from the LUMO orbital graph of C (σ(p_y_))-Pt (σ(d_xy_))-X (σ(p_X_)) that the φ^2^ of Pt increase and the φ^2^ of C and X decrease.

In the course of the formation of H_2_, the positive charge of H diminishes, indicating that it may be an HT mechanism. Corresponding to this, electron density has been transferred from methane to hydrogen, as shown in the HOMO orbital graph in Figure 5. As the transferred electron density takes the same route as that of the concurrently transferred hydrogen atom, it can be described as a hydride transfer mechanism. For different halogens, the change of charges is the same, that is to say, in terms of rate controlling step, different halogen ligands have no significant effect on the process of elimination of H_2_.

In sum, in the eliminations of HX, the mechanisms are different. The transfer form of H is from proton to atom, last to hydride. The reason is mainly due to the electronegativity of halogens, while for the loss of H_2_, the transfer of H is in the form of hydride for all the X ligands.

### 3.4 Analysis of interaction between complexes

**FIGURE 6:**
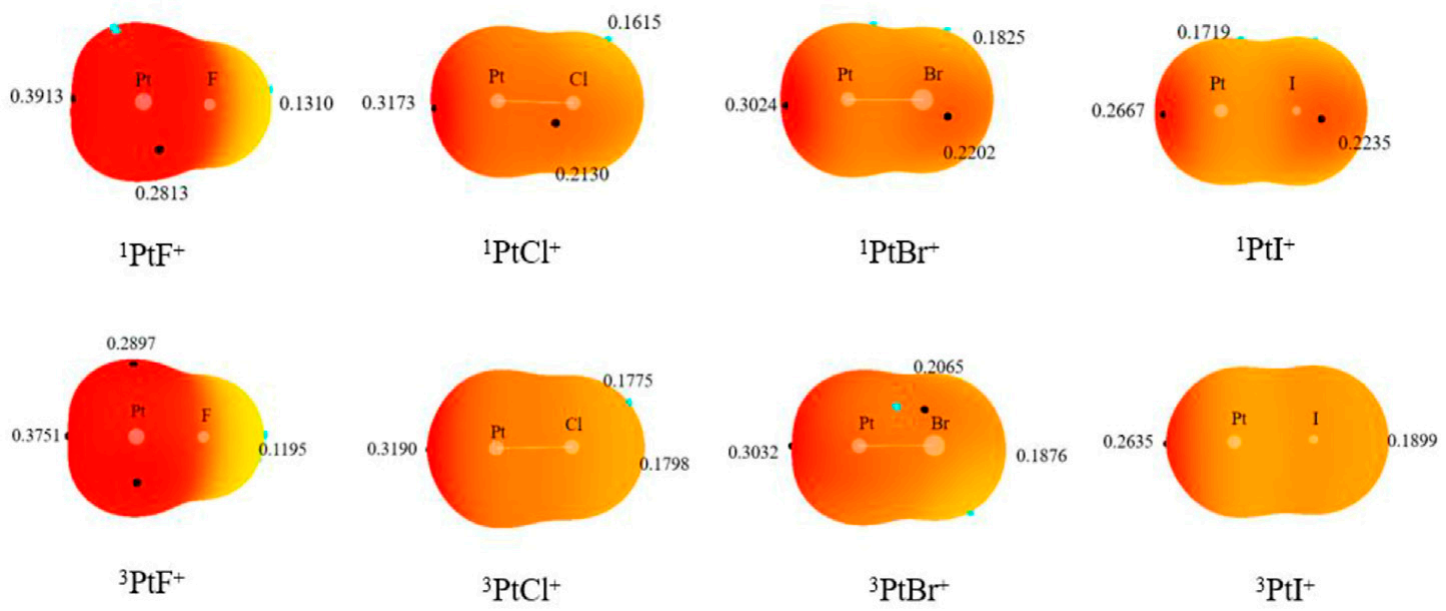
Molecular electrostatic potential (MEP) maps on the 0.001 a. u. isodensity surface of the monomers.

To further investigate the mechanisms, the interactions between the complexes in the reactions were also discussed. The MEP diagrams of singlet and triplet PtX^+^ on the 0.001 a. u. isodensity surface are displayed in Figure 6. Since the complexes have positive charge, the overall electrostatic potential is in the red region. There is a deep red region (σ-hole) along the Pt-F axis around the Pt^+^, which correspond to the site where carbon atoms attack PtX^+^. It is consistent with the previously optimized structure. In addition, the σ-hole strength decreases in the order PtF^+^> PtCl^+^> PtBr^+^> PtI^+^ owing to the different electron-withdrawing ability among the halogen.

In the AIM theory, electron density at the bond critical point (BCP) is one of the indicators of interaction strength. The electron density, Laplacian, and total energy density at the XPt-CH_4_ (X = F, Cl, Br, I) complexes are listed in Table 4. The values of the density at the BCP lie in the range around 0.07 a. u., Laplacian is positive and energy density is negative in the methane complexes, which indicates that there exhibits a property of a partially covalent interaction. The charge transfer from CH_4_ to PtX^+^ decreases in the order PtF^+^> PtCl^+^> PtBr^+^> PtI^+^, which is consistent with the σ-hole strength of PtX^+^. The charge transfer and the interaction energy of methane complexes in the potential energy surface have similar changes.

**TABLE 4:** Electron density (ρ, a.u.), Laplacian (▽^2^ρ, a.u.), energy density (H, a.u.), intermolecular distance (R, Å), and charge transfer (CT, e) at the XPt-CH_4_ (X = F, Cl, Br, I) complexes.

Complexes	ρ	▽^2^ρ	H	R	CT
FPt-CH_4_	0.0690	0.2009	−0.0143	2.305	0.2101
ClPt-CH_4_	0.0727	0.1943	−0.0163	2.284	0.2111
BrPt-CH_4_	0.0685	0.1886	−0.0140	2.315	0.1927
IPt-CH_4_	0.0671	0.1888	−0.0133	2.327	0.1764

Table 5 shows the electron density, Laplacian, and total energy density at the complexes before the expulsions of HX or H_2_. It is generally believed that the greater the electron density between two atoms in a composite, the more concentrated the charge between the two atoms, which also means that the bond between these two atoms has a stronger tendency (Kraka and Cremer, 1990; Alkorta et al., 1998). As shown in Table 5, for all the complexes before dissociating the H_2_ or HX molecule, Laplacian is positive and energy density is negative, indicating that there exists a partially covalent interaction between H_2_ or HX molecule and Pt atom. For the same complex, the values of **ρ**, **▽** ^**2**^ **ρ**, and energy density are obviously stronger in the singlet state than those in the triplet state, indicating a stronger interaction between the Pt and the H_2_ or HX molecule in the singlet state, and more energy is required to expel H_2_ or HX, which is consistent with the energy barrier of the reaction in the potential energy surfaces. Through the analysis of the interaction between atoms in the transition metal–ligand complex, the reaction path can be better explained.

**TABLE 5:** Electron density (ρ, a.u.), Laplacian (▽^2^ρ, a.u.), energy density (H, a.u.), and charge transfer (CT, e) in the PtXCH_2_-H_2_ and PtCH_3_-HX (X = F, Cl, Br, I) complexes.

	ρ	▽^2^ρ	H	CT	ρ	▽^2^ρ	H	CT
	Singlet				Triplet			
PtFCH_2_-H_2_	0.1221	0.3547	−0.0499	0.2136	0.0683	0.2060	−0.0122	0.1537
PtClCH_2_-H_2_	0.1088	0.3689	−0.0362	0.1873	0.0720	0.2209	−0.0141	0.1451
PtBrCH_2_-H_2_	0.1037	0.3555	−0.0325	0.1749	0.0621	0.2265	−0.0094	0.1081
PtICH_2_-H_2_	0.1120	0.3126	−0.0402	0.1923	0.0691	0.2589	−0.0121	0.1132
PtCH_3_-HF	0.0496	0.2738	−0.0004	0.0655	0.0404	0.2029	0.0002	0.0486
PtCH_3_-HCl	0.0833	0.2468	−0.0198	0.2990	0.0536	0.1550	−0.0082	0.2091
PtCH_3_-HBr	0.0866	0.1773	−0.0269	0.3817	0.0574	0.1281	−0.0118	0.2550
PtCH_3_-HI	0.0690	0.0774	−0.0210	0.4690	0.0489	0.0739	−0.0099	0.3503

From Table 5, one also can see that for the complexes before H_2_ was removed, the values of charge transfer (CT) are between 0.17 e and 0.21 e, and the differences between PtXCH_2_-H_2_ (X = F, Cl, Br, I) are small. However, for the complexes before HX was expelled, the values of charge transfer increased from PtCH_3_-HF to PtCH_3_-HI, which was generally consistent with the interaction energy. Namely, the complexes before the expulsions of HI had larger interaction energy than the others.

### 3.5 Comparisons with the reactions of NiX^+^ (X = F, Cl, Br, I) + CH_4_

The reactions of NiX^+^ (X = F, Cl, Br, I) and methane have been investigated at the B3LYP level of theory by Schlangen *et al.* (Schlangen et al., 2007; Schlangen and Schwarz, 2008). They reported that NiF^+^ is the only nickel-halide complex capable of activating methane for NiX^+^ (X = F, Cl, Br, I). The driving force of the reaction NiF^+^ with methane is provided by the exceptionally high stability of HF (Schlangen et al., 2007). In the present study, we found that the PtX^+^ (X = F, Cl, Br) can bring about thermal activation of methane to loss HX decreasing in going as the sequence X = F, Cl, Br, and to loss H_2_ increasing in the reverse sequence.

The reactions of NiF^+^ and their third-row congeners PtX^+^ with methane have many features in common; whereas, fundamental differences exist with regard to the details of the potential energy surfaces and, thus, to actual reaction mechanisms. As reported by Schlangen and Schwarz (2008), for the NiF^+^ and methane systems studied, the energetically most favored variant corresponds to an σ-complex-assisted metathesis (σ-CAM). First, the reactions start with the formation of the encounter complex NiF(CH_4_)^+^, and then, the molecule HF is directly eliminated *via* a multicenter transition state to obtain the product complex, Ni(CH_3_)(HF)^+^. However, in the PtX^+^/CH_4_ systems, oxidative addition/reductive elimination (OA/RE) is operative. After the formation of the complex PtX (CH_4_)^+^, the next step is the cleaving of the C-H bond (oxidative addition), resulting in the insertion product PtHX (CH_3_)^+^, and then a reductive elimination step to form an HX molecule complex is obtained, that is, the H and X rearrange to form an HX molecule electrostatically bound to Pt to obtained the ^1^Pt (CH_3_)(HX)^+^.

Another difference in the reaction mechanisms is the potential energy surfaces. In the NiF^+^/CH_4_ system, the exothermic ligand exchange proceeds adiabatically only on the one potential energy surface, whereas the reaction of PtX^+^/CH_4_ needs a curve crossing, that is to say, it is a two-state reactivity. Otherwise, the NiF^+^/CH_4_ system proceeds on the high-spin ground triplet state, while the reaction of PtX^+^ with methane takes place more easily along the low-spin potential energy surface. As for the reasons for the differences, Schlangen and Schwarz (2008) have reported that the strongly electron-withdrawing F substituent reduces the electron density at the Ni-center and, thus, decreases the repulsive interaction; therefore, the reaction can proceed on the high-spin ground state. Based on this point, we calculated the Mullikan charges of the M-atom (M = Ni, Pt) in the systems MX^+^/CH_4_. The results indicate that the Mullikan charges of the Ni-atom in the NiF^+^/CH_4_ system increase 0.198, while the Pt-atom in the reaction PtF^+^/CH_4_ increases only 0.082.

## 4 Conclusion

The gas-phase ion-molecule reactions of PtX^+^ cations (X = F, Cl, Br, I) with methane have been investigated theoretically at the DFT (B3LYP) level, considering both the low- and high-spin potential energy surfaces. All reactions fall into two major categories: 1) reactions involving Pt-X bond cleavage to expulse HX and 2) bond activation of CH_4_ without obvious occurrence of Pt-X bond cleavage to loss H_2_. In the elimination of HX (X = F, Cl, Br, I), this reaction channel decreases in going as the sequence: X = F, Cl, Br, I. A reversed trend is observed in the losses of small closed-shell molecule H_2_ for X = F, Cl, Br, while it is not favorable for PtI^+^ in the loss of either HI or H_2_. The reason for the reactivity along with the abovementioned trends can be explained by the electronegative character of X.

In the eliminations of HX, the transfer form of H is from proton to atom, last to hydride, and the mechanisms are from PECT to HAT, last to HT for the sequence of X = F, Cl, Br, I. One reason is mainly due to the electronegativity of halogens. Otherwise, the mechanisms of HX eliminations also can be explained by the analysis of Frontier Molecular Orbitals, while for the loss of H_2_, the transfer of H is in the form of hydride for all the X ligands.

The charge transfer from CH_4_ to PtX^+^ decreases in the order PtF^+^> PtCl^+^> PtBr^+^> PtI^+^, which is consistent with the σ-hole strength of PtX^+^. For the same complex, the values of ρ, ▽^2^ρ, and energy density are obviously stronger in the singlet state than those in the triplet state, indicating a stronger interaction between the Pt and the H_2_ or HX molecule in the singlet state, and more energy is required to expel H_2_ or HX, which is consistent with the energy barrier of the reaction in the potential energy surfaces. The differences in charge transfer between PtXCH_2_-H_2_ (X = F, Cl, Br, I) for the complexes before H_2_ is removed are small. However, for the complexes before HX is expelled, the values of charge transfer increase from PtCH_3_-HF to PtCH_3_-HI, namely, the complexes before the expulsions of HI have larger interaction energy than the others. Through the analysis of the interaction between atoms in the transition metal ligand complex, the reaction path can be better explained.

## Data Availability

The original contributions presented in the study are included in the article/Supplementary Material; further inquiries can be directed to the corresponding author.

## References

[B1] Achatz U. Berg C. Joos S. Fox B. S. Beyer M. K. Niedner-Schatteburg G. (2000). Methane activation by platinum cluster ions in the gas phase: Effects of cluster charge on the Pt_4_ tetramer. Chem. Phys. Lett. 320, 53–58. 10.1016/S0009-2614(00)00179-2

[B2] Alkorta I. Rozas I. Elguero J. (1998). Charge-transfer complexes between dihalogen compounds and electron donors. J. Phys. Chem. A 102, 9278–9285. 10.1021/jp982251o

[B3] Andrae D. Haeussermann U. Dolg M. Stoll H. Preuss H. (1990). Energy-adjusted*ab initio* pseudopotentials for the second and third row transition elements. Theor. Chim. Acta 77, 123–141. 10.1007/BF01114537

[B4] Bader R. F. W. (2000). AIM2000 Program. Hamilton, Canada: McMaster University. v. 2.0.

[B5] Balcells D. Clot E. Eisenstein O. (2010). C-H bond activation in transition metal species from a computational perspective. Chem. Rev. 110, 749–823. 10.1021/cr900315k 20067255

[B6] Becke A. D. (1988). Density-functional exchange-energy approximation with correct asymptotic behavior. Phys. Rev. A . Coll. Park. 38, 3098–3100. 10.1103/PhysRevA.38.3098 9900728

[B7] Becke A. D. (1993). Density‐functional thermochemistry. III. The role of exact exchange. J. Chem. Phys. 98, 5648–5652. 10.1063/1.464913

[B8] Bulat F. A. Toro-Labbé A. Brinck T. Murray J. S. Politzer P. (2010). Quantitative analysis of molecular surfaces: Areas, volumes, electrostatic potentials and average local ionization energies. J. Mol. Model. 16, 1679–1691. 10.1007/s00894-010-0692-x 20361346

[B9] Chen Q. Chen H. P. Kais S. Freiser B. S. (1997). Gas-phase reactions of Fe(CH_2_O)^+^ and Fe(CH_2_S)^+^ with small alkanes: An experimental and theoretical study. J. Am. Chem. Soc. 119, 12879–12888. 10.1021/ja964234n

[B10] Dede Y. Zhang X. H. Schlangen M. Schwarz H. Baik M. H. (2009). A redox non-innocent ligand controls the life time of a reactive quartet excited state-an MCSCF study of [Ni(H)(OH)]^+^ . J. Am. Chem. Soc. 131, 12634–12642. 10.1021/ja902093f 19670859

[B11] Dietl N. Schlangen M. Schwarz H. (2012). Thermal hydrogen‐atom transfer from methane: The role of radicals and spin states in oxo‐cluster chemistry. Angew. Chem. Int. Ed. 51, 5544–5555. 10.1002/anie.201108363 22431300

[B12] Dobereine G. E. Crabtree R. H. (2010). Dehydrogenation as a substrate-activating strategy in homogeneous transition-metal catalysis. Chem. Rev. 110, 681–703. 10.1021/cr900202j 19938813

[B13] Dubois M. R. (1989). Catalytic applications of transition-metal complexes with sulfide ligands. Chem. Rev. 89, 1–9. 10.1021/cr00091a001

[B14] Elkind J. L. Armentrout P. B. (1987). State-specific reactions of atomic transition-metal ions with H_2_, HD, and D_2_: Effects of d orbitals on chemistry. J. Phys. Chem. 91, 2037–2045. 10.1021/j100292a012

[B15] Eller K. Schwarz H. (1991). Organometallic chemistry in the gas phase. Chem. Rev. 91, 1121–1177. 10.1021/cr00006a002

[B16] Frisch M. J. Trucks G. W. Schlegel H. B. Scuseria G. E. Robb M. A. Cheeseman J. R. (2009). Gaussian 09. revision A. 02. Wallingford CT: Gaussian, Inc.

[B17] Geng C. Y. Li J. L. Weiske T. Schlangen M. Shaik S. Schwarz H. (2017). Electrostatic and charge-induced methane activation by a concerted double C-H bond insertion. J. Am. Chem. Soc. 139, 1684–1689. 10.1021/jacs.6b12514 28051294

[B18] Gonzalez C. Schlegel H. B. (1989). An improved algorithm for reaction path following. J. Chem. Phys. 90, 2154–2161. 10.1063/1.456010

[B19] Halle L. F. Armentrout P. B. Beauchamp J. L. (1982). Ion beam studies of the reactions of group VIII metal ions with alkanes: Correlation of thermochemical properties and reactivity. Organometallics 1, 963–968. 10.1021/om00067a012

[B20] Harvey J. N. (2007). Understanding the kinetics of spin-forbidden chemical reactions. Phys. Chem. Chem. Phys. 9, 331–343. 10.1039/b614390c 17199148

[B21] Howell J. A. S. Burkinshaw P. M. (1983). Ligand substitution reactions at low-valent four-five-and six-coordinate transition metal centers. Chem. Rev. 83, 557–599. 10.1021/cr00057a005

[B22] Irikura K. K. Beauchamp J. L. (1991b). Electronic structure considerations for methane activation by third-row transition-metal ions. J. Phys. Chem. 95, 8344–8351. 10.1021/j100174a057

[B23] Irikura K. K. Beauchamp J. L. (1991a). Methane oligomerization in the gas phase by third-row transition-metal ions. J. Am. Chem. Soc. 113, 2769–2770. 10.1021/ja00007a070

[B24] Irikura K. K. Beauchamp J. L. (1989). Osmium tetroxide and its fragment ions in the gas phase: Reactivity with hydrocarbons and small molecules. J. Am. Chem. Soc. 111, 75–85. 10.1021/ja00183a014

[B25] Jana R. Pathak T. P. Sigman M. S. (2011). Advances in transition metal (Pd, Ni, Fe)-catalyzed cross-coupling reactions using alkyl-organometallics as reaction partners. Chem. Rev. 111, 1417–1492. 10.1021/cr100327p 21319862 PMC3075866

[B26] Kraka E. Cremer D. (1990). Chemical implication of local features of the electron density distribution. Concept Chem. Bond, 116, 453–542. Maksic, ZB, Ed. 10.1007/978-3-642-61277-0_12

[B27] Lawson Daku L. M. Aquilante F. Robinson T. W. Hauser A. (2012). Accurate spin-state energetics of transition metal complexes. 1. CCSD(T), CASPT2, and DFT study of [M(NCH)_6_]^2+^ (M = Fe, Co). J. Chem. Theory Comput. 8, 4216–4231. 10.1021/ct300592w 26605585

[B28] Lee C. Yang W. Parr R. G. (1988). Development of the Colle-Salvetti correlation-energy formula into a functional of the electron density. Phys. Rev. B 37, 785–789. 10.1103/PhysRevB.37.785 9944570

[B29] Li J. L. Zhou S. D. Schlangen M. Weiske T. Schwarz H. (2016a). Hidden hydride transfer as a decisive mechanistic step in the reactions of the unligated gold carbide [AuC]^+^ with methane under ambient conditions. Angew. Chem. Int. Ed. 55, 13072–13075. 10.1002/anie.201606707 27647692

[B30] Li J. L. Zhou S. D. Zhang J. Schlangen M. Usharani D. Shaik S. (2016b). Mechanistic variants in gas-phase metal-oxide mediated activation of methane at ambient conditions. J. Am. Chem. Soc. 138, 11368–11377. 10.1021/jacs.6b07246 27518766

[B31] Li J. L. Zhou S. D. Zhang J. Schlangen M. Weiske T. Usharani D. (2016c). Electronic origins of the variable efficiency of room-temperature methane activation by homo-and heteronuclear cluster oxide cations [XYO_2_]^+^ (X, Y= Al, Si, Mg): Competition between proton-coupled electron transfer and hydrogen-atom transfer. J. Am. Chem. Soc. 138, 7973–7981. 10.1021/jacs.6b03798 27241233

[B32] Li W. Q. Geng Z. Y. Wang Y. C. Yan P. J. Zhang X. Wang Z. (2009). Density functional theory studies of thermal activation of methane by MH^+^ (M= Ru, Rh, and Pd). J. Phys. Chem. A 113, 1807–1812. 10.1021/jp808830c 19199493

[B33] Liu G. X. Zhu Z. G. Ciborowski S. M. Ariyarathna I. R. Miliordos E. Bowen K. H. (2019). Selective activation of the C-H bond in methane by single platinum atomic anions. Angew. Chem. Int. Ed. 58, 7773–7777. 10.1002/anie.201903252 30968506

[B34] Mandich M. L. Steigerwald M. L. Reents W. D. (1986). The effects of chloro substitution on the electronic structure of ClCr^+^, ClMn^+^, and ClFe^+^ and their reactivity with small alkanes. J. Am. Chem. Soc. 108, 6197–6202. 10.1021/ja00280a015

[B35] Mazurek U. Schröder D. Schwarz H. (1998). Generation and reactivity of chromium fluoride cations (CrF_n_ ^+^, n= 0-4) in the gas phase. Collect. Czech. Chem. Commun. 63, 1498–1512. 10.1135/cccc19981498

[B36] Michael B. Christoph R. Dimitrios A. P. Thomas B. Frank N. (2008). Geometries of third-row transition-metal complexes from density-functional theory. J. Chem. Theory Comput. 4, 1449–1459. 10.1021/ct800172j 26621431

[B37] Musaev D. G. Koga N. Morokuma K. (1993). *Ab initio* molecular orbital study of the electronic and geometric structure of RhCH_2_ ^+^ and the reaction mechanism: RhCH_2_ ^+^ + H_2_. → Rh^+^ + CH_4_ . J. Phys. Chem. 97, 4064–4075. 10.1021/j100118a022

[B38] Musaev D. G. Morokuma K. (1994). *Ab initio* molecular orbital study of the molecular and electronic structure of FeCH_2_ ^+^ and of the reaction mechanism of FeCH_2_ ^+^ + H_2_ . J. Chem. Phys. 101, 10697–10707. 10.1063/1.467883

[B39] Ohanessian G. Brusich M. J. Goddard W. A. III (1990). Theoretical study of transition-metal hydrides. 5. Hafnium to mercury (HfH^+^ through HgH^+^), barium and lanthanum (BaH^+^ and LaH^+^). J. Am. Chem. Soc. 112, 7179–7189. 10.1021/ja00176a016

[B40] Perdew J. P. (1986a). Density-functional approximation for the correlation energy of the inhomogeneous electron gas. Phys. Rev. B 33, 8822–8824. 10.1103/PhysRevB.33.8822 9938299

[B41] Perdew J. P. (1986b). Erratum: Density-functional approximation for the correlation energy of the inhomogeneous electron gas. Phys. Rev. B 34, 7406. 10.1103/PhysRevB.34.7406 9949100

[B42] Poli R. Harvey J. N. (2003). Spin forbidden chemical reactions of transition metal compounds. New ideas and new computational challenges. Chem. Soc. Rev. 32, 1–8. 10.1039/b200675h 12596540

[B43] Rodgers M. T. Stanley J. R. Amunugama R. (2000). Periodic trends in the binding of metal ions to pyridine studied by threshold collision-induced dissociation and density functional theory. J. Am. Chem. Soc. 122, 10969–10978. 10.1021/ja0027923

[B44] Roithova J. Schröder D. (2010). Selective activation of alkanes by gas-phase metal ions. Chem. Rev. 110, 1170–1211. 10.1021/cr900183p 20041696

[B45] Schilling J. B. Goddard W. A. III Beauchamp J. L. (1986). Theoretical studies of transition-metal hydrides. 1. Bond energies for MH^+^ with M= Ca, Sc, Ti, V, Cr, Mn, Fe, Co, Ni, Cu, and Zn. J. Am. Chem. Soc. 108, 582–584. 10.1021/ja00264a004

[B46] Schilling J. B. Goddard W. A. III Beauchamp J. L. (1987). Theoretical studies of transition-metal hydrides. 2. CaH^+^ through ZnH^+^ . J. Phys. Chem. 91, 5616–5623. 10.1021/j100306a024

[B47] Schilling J. B. Goddard W. A. III Beauchamp J. L. (1987). Theoretical studies of transition-metal hydrides. 3. SrH^+^ through CdH^+^ . J. Am. Chem. Soc. 109, 5565–5573. 10.1021/ja00253a001

[B48] Schlangen M. Schröder D. Schwarz H. (2007). Ligand and substrate effects in gas‐phase reactions of NiX^+^/RH couples (X= F, Cl, Br, I; R= CH_3_, C_2_H_5_, nC_3_H_7_, nC_4_H_9_). Chem. Eur. J. 13, 6810–6816. 10.1002/chem.200700506 17591727

[B49] Schlangen M. Schröder D. Schwarz H. (2007). Pronounced ligand effects and the role of formal oxidation states in the nickel-mediated thermal activation of methane. Angew. Chem. Int. Ed. 46, 1641–1644. 10.1002/anie.200603266 17262870

[B50] Schlangen M. Schwarz H. (2008). Ligand effects on the mechanisms of thermal bond activation in the gas‐phase reactions NiX^+^/CH_4_ → Ni(CH_3_)^+^/HX (X= H, CH_3_, OH, F). Short Communication. Helv. Chim. Acta 91, 2203–2210. 10.1002/hlca.200890238

[B51] Schröder D. Hrusak J. Schwarz H. (1993). Ligand effects on the reactivity of iron (II) cations FeX^+^ in the gas phase. Berichte Bunsenges. fur Phys. Chem. 97, 1085–1090. 10.1002/bbpc.19930970904

[B52] Schröder D. Schwarz H. (2005). Activation of methane by gaseous platinum (II) ions PtX^+^ (X= H, Cl, Br, CHO). Can. J. Chem. 83, 1936–1940. 10.1139/v05-217

[B53] Schultz R. H. Elkind J. L. Armentrout P. B. (1988). Electronic effects in C-H and C-C bond activation. State-specific reactions of Fe^+^ (^6^D, ^4^F) with methane, ethane, and propane. J. Am. Chem. Soc. 110, 411–423. 10.1021/ja00210a017

[B54] Schwarz H. Navarrete P. G. Li J. L. Schlangen M. Sun X. Y. Weiske T. (2017). Unexpected mechanistic variants in the thermal gas-phase activation of methane. Organometallics 36, 8–17. 10.1021/acs.organomet.6b00372

[B55] Schwarz H. Shaik S. Li J. L. (2017). Electronic effects on room-temperature, gas-phase C-H Bond activations by cluster oxides and metal carbides: The methane challenge. J. Am. Chem. Soc. 139, 17201–17212. 10.1021/jacs.7b10139 29112810

[B56] Sun X. Y. Zhou S. D. Schlangen M. Schwarz H. (2016). Efficient room‐temperature methane activation by the closed‐shell, metal‐free cluster [OSiOH]^+^: A novel mechanistic variant. Chem. Eur. J. 22, 14257–14263. 10.1002/chem.201601981 27515768

[B57] Tolbert M. A. Beauchamp J. L. (1986). Homolytic and heterolytic bond dissociation energies of the second row group 8, 9, and 10 diatomic transition-metal hydrides: Correlation with electronic structure. J. Phys. Chem. 90, 5015–5022. 10.1021/j100412a029

[B58] Tolbert M. A. Mandich M. L. Halle L. F. Beauchamp J. L. (1986). Activation of alkanes by ruthenium, rhodium, and palladium ions in the gas phase: Striking differences in reactivity of first-and second-row metal ions. J. Am. Chem. Soc. 108, 5675–5683. 10.1021/ja00279a003 22175311

[B59] Vargas A. Krivokapic I. Hauser A. Lawson Daku L. M. (2013). Towards accurate estimates of the spin-state energetics of spin-crossover complexes within density functional theory: A comparative case study of cobalt(II) complexes. Phys. Chem. Chem. Phys. 15, 3752–3763. 10.1039/c3cp44336a 23389801

[B60] Wang X. F. Andrews L. (2009). Infrared spectra and theoretical calculations for Fe, Ru, and Os metal hydrides and dihydrogen complexes. J. Phys. Chem. A 113, 551–563. 10.1021/jp806845h 19099441

[B61] Wesendrup R. Schroder D. Schwarz H. (1994). Catalytic Pt^+^‐mediated oxidation of methane by molecular oxygen in the gas phase. Angew. Chem. Int. Ed. Engl. 33, 1174–1176. 10.1002/anie.199411741

[B62] Westerberg J. Blomberg M. R. A. (1998). Methane activation by naked Rh^+^ atoms. A theoretical study. J. Phys. Chem. A 102, 7303–7307. 10.1021/jp981291p

[B63] Wheeler O. W. Michelle S. Gao A. Bakker J. M. Armentrout P. B. (2016). Activation of C-H bonds in Pt^+^ + x CH_4_ reactions, where x= 1-4: Identification of the platinum dimethyl cation. J. Phys. Chem. A 120, 6216–6227. 10.1021/acs.jpca.6b05361 27438025

[B64] Ye S. Neese F. (2010). Accurate modeling of spin-state energetics in spin-crossover systems with modern density functional theory. Inorg. Chem. 49, 772–774. 10.1021/ic902365a 20050628

[B65] Yue L. Li J. L. Zhou S. D. Sun X. Y. Schlangen M. Shaik S. (2017). Control of product distribution and mechanism by ligation and electric field in the thermal activation of methane. Angew. Chem. Int. Ed. 56, 10219–10223. 10.1002/anie.201703485 28544127

[B66] Zhang Q. Bowers M. T. (2004). Activation of methane by MH^+^ (M= Fe, Co, and Ni): A combined mass spectrometric and DFT study. J. Phys. Chem. A 108, 9755–9761. 10.1021/jp047943t

[B67] Zhao Y. Truhlar D. G. (2008). Density functionals with broad applicability in chemistry. Acc. Chem. Res. 41, 157–167. 10.1021/ar700111a 18186612

[B68] Zhao Y. Truhlar D. G. (2008). The M06 suite of density functionals for main group thermochemistry, thermochemical kinetics, noncovalent interactions, excited states, and transition elements: Two new functionals and systematic testing of four M06-class functionals and 12 other functionals. Theor. Chem. Acc. 120, 215–241. 10.1007/s00214-007-0310-x

[B69] Zhou S. D. Firouzbakht M. Schlangen M. Kaupp M. Schwarz H. (2017a). On the electronic origin of remarkable ligand effects on the reactivities of [NiL]^+^ complexes (L= C_6_H_5_, C_5_H_4_N, CN) towards methane. Chem. Eur. J. 23, 14430–14433. 10.1002/chem.201703767 28865112

[B70] Zhou S. D. Li J. L. Firouzbakht M. Schlangen M. Schwarz H. (2017b). Sequential gas-phase activation of carbon dioxide and methane by [Re(CO)_2_]^+^: The sequence of events matters. J. Am. Chem. Soc. 139, 6169–6176. 10.1021/jacs.7b01255 28403605

[B71] Zhou S. D. Li J. L. Schlangen M. Schwarz H. (2016). Thermal dehydrogenation of methane by [ReN]^+^ . Angew. Chem. Int. Ed. 55, 14863–14866. 10.1002/anie.201607960 27726248

[B72] Zhou S. D. Schlangen M. Schwarz H. (2017c). Spin‐Selective, Competitive hydrogen‐atom transfer versus CH_2_O‐generation from the CH_4_/[ReO_4_]^+^ couple at ambient conditions. Chem. Eur. J. 23, 17469–17472. 10.1002/chem.201704892 29095534

