# Supplementary material for: CH4 activation by PtX+ (X = F, Cl, Br, I)
Source: Front Chem. 2022 Sep 26;10:1027465. doi: 10.3389/fchem.2022.1027465 (PMC9548706; doi:10.3389/fchem.2022.1027465)
Supplement: Supplementary file 1 [file DataSheet1.PDF]

CH_4 Activation by PtX^+ (X =F, Cl, Br, I)

Jin Zhao‡, Lingxi Qi‡, Wenzuo Li*, Jianbo Cheng, Qingzhong Li* and Shaoli Liu*

College of Chemisty and Chemical Engineering, Yantai University,

Yantai 264005, P. R. China

Description: Geometry optimization for all of the reactants, intermediates, transition states, and products were carried out with the 6-311+G** basis set for carbon and hydrogen, also for F, Cl, Br, and I atoms of the reactions investigated. The Stuttgart/Dresden relativistic effective core potentials (ECP) of SDD were adopted to describe the metal Pt and the halogen I. Bond lengths are in angstroms and bond angles in degrees.

In this Supporting Information files, the valence NBO populations for the 6s/5d/6p orbitals of Pt and the Natural population analysis (NPA) charge of the related atoms in the related intermediates in the reaction of PtX^+ (X = F, Cl, Br, I) + CH_4 are listed in the Table S1 - S4, the single point energy calculation of the reaction PtX+ (X = F, Cl, Br, I) + CH_4 in the singlet and triplet state at the CCSD(T)/aug-cc-pvtz level in the Table S5, the imaginary frequencies of the transition states in the PESs are listed in the Table S6, the optimized geometries for the stationary points of the reaction PtX^+ (X = F, Cl, Br, I) + CH_4 in the singlet and triplet state (bond lengths in angstroms and bond angles in degrees) are listed in Figure S1 - S8.

Through the verification of single point energy, we found that the calculation results obtained by the method of CCSD(T) is basically similar to the previous trend of the potential energy surface, except that the last step in eliminate hydrogen and hydride does not conform to the experimental results, so the B3LYP is practicable for modeling this class of reactions such as PtX^+/CH_4 systems.

Table S1. Valence NBO populations for the 6s/5d/6p orbitals of Pt and the Natural population analysis (NPA) charge of the related atoms in the related intermediates in the reaction of PtF^+ + CH_4

species	NBO		NPA charge					
	singlet	triplet	singlet			triplet		
			Pt	F	C	Pt	F	C
PtF^+	0.04/8.57/0.02	0.21/8.43/0.01	1.37	-0.37		1.36	-0.36	
$PtF(CH_4)^+$		0.38/8.39/0.02				1.20	-0.42	-0.84
TS1		0.59/8.346/0.01				1.05	-0.37	-0.74
$PtHF(CH_3)^+$	0.53/8.49/0.01	0.62/8.34/0.01	0.99	-0.38	-0.52	1.03	-0.37	-0.69
TS2	0.42/8.73/0.01	0.54/8.42/0.01	0.85	-0.40	-0.52	1.03	-0.43	-0.65
$Pt(CH_3)(HF)^+$	0.15/9.01/0.01	0.57/8.49/0.01	0.84	-0.54	-0.54	0.94	-0.56	-0.70
$Pt(CH_3)^+$+HF	0.07/9.05/0.01	0.64/8.44/0.01						
TS2-H_2	0.67/8.52/0.03	0.64/8.46/0.03	0.79	-0.52	-0.13	0.87	-0.39	-0.39
$PtH_2F(CH_2)^+$	0.66/8.52/0.01	0.64/8.49/0.03	0.80	-0.55	-0.10	0.85	-0.39	-0.37
TS3-H_2	0.62/8.57/0.01	0.57/8.50/0.02	0.80	-0.54	0.02	0.91	-0.40	-0.33
$PtF(CH_2)(H_2)^+$	0.57/8.57/0.02	0.56/8.37/0.02	0.84	-0.55	0.07	1.04	-0.43	-0.21
$PtF(CH_2)^+$+H_2	0.51/8.49/0.01	0.50/8.32/0.01	1.00	-0.43	-0.01	1.16	-0.41	-0.21

Table S2. Valence NBO populations for the 6s/5d/6p orbitals of Pt and the Natural population analysis (NPA) charge of the related atoms in the related intermediates in the reaction of $PtCl^+$ + CH_4

species	NBO		NPA charge					
	singlet	triplet	singlet			triplet		
			Pt	Cl	C	Pt	Cl	C
$PtCl^+$	0.39/8.67/0.01	0.45/8.61/0.01	0.92	0.08		0.92	0.08	
$PtCl(CH_4)^+$		0.66/8.56/0.02				0.75	0.04	-0.87
TS1		0.65/8.62/0.01				0.72	-0.002	-0.72
$PtHCl(CH_3)^+$	0.59/8.71/0.01	0.59/8.61/0.01	0.68	-0.08	-0.52	0.80	-0.11	-0.71-
TS2	0.43/8.93/0.01	0.65/8.57/0.01	0.64	-0.02	-0.52	0.76	-0.04	-0.69
$Pt(CH_3)(HCl)^+$	0.36/9.03/0.01	0.59/8.61/0.01	0.60	-0.02	-0.55	0.80	-0.11	-0.71
TS2-H_2	0.72/8.69/0.02	0.65/8.76/0.02	0.56	-0.25	-0.15	0.57	0.01	-0.44
$PtH_2Cl(CH_2)^+$	0.72/8.72/0.03	0.67/8.75/0.03	0.58	-0.28	-0.12	0.56	0.05	-0.43
TS3-H_2	0.68/8.70/0.01	0.63/8.71/0.02	0.58	-0.27	-0.02	0.63	-0.04	-0.35
$PtCl(CH_2)(H_2)^+$	0.62/8.71/0.02	0.63/8.59/0.02	0.64	-0.26	0.02	0.75	-0.08	-0.25
$PtCl(CH_2)^+$+H_2	0.60/8.66/0.01	0.67/8.53/0.02	0.74	-0.14	-0.03	0.78	-0.03	-0.18

Table S3. Valence NBO populations for the 6s/5d/6p orbitals of Pt and the Natural population analysis (NPA) charge of the related atoms in the related intermediates in the reaction of $PtBr^+$ + CH_4

species	NBO		NPA charge					
	singlet	triplet	singlet			triplet		
			Pt	Br	C	Pt	Br	C
$PtBr^+$	0.46/8.77/0.01	0.48/8.71/0.01	0.76	0.24		0.79	0.21	
$PtBr(CH_4)^+$		0.68/8.67/0.02				0.62	0.19	-0.87
TS1		0.66/8.70/0.01				0.61	0.13	-0.72
$PtHBr(CH_3)^+$	0.41/9.05/0.01	0.67/8.70/0.01	0.53	0.13	-0.56	0.61	0.13	-0.69
TS2	0.41/9.01/0.01	0.65/8.62/0.01	0.57	0.12	-0.54	0.71	0.09	-0.70
$Pt(CH_3)(HBr)^+$	0.41/9.05/0.01	0.79/8.45/0.01	0.53	0.13	-0.56	0.75	0.004	-0.70
TS2-H_2	0.73/8.72/0.03	0.67/8.82/0.02	0.51	-0.15	-0.17	0.48	0.14	-0.44
$PtH_2Br(CH_2)^+$	0.74/8.69/0.03	0.69/8.80/0.03	0.53	-0.19	-0.14	0.48	0.17	-0.43
TS3-H_2	0.70/8.73/0.01	0.64/8.81/0.02	0.53	-0.18	-0.05	0.51	0.13	-0.36
$PtBr(CH_2)(H_2)^+$	0.64/8.74/0.01	0.66/8.69/0.03	0.59	-0.16	-0.004	0.62	0.10	-0.23
$PtBr(CH_2)^+$+H_2	0.63/8.70/0.01	0.69/8.62/0.02	0.66	-0.04	-0.05	0.68	0.10	-0.19

Table S4. Valence NBO populations for the 6s/5d/6p orbitals of Pt and the Natural population analysis (NPA) charge of the related atoms in the related intermediates in the reaction of PtI^+ + CH_4

species	NBO		NPA charge					
	singlet	triplet	singlet			triplet		
			Pt	I	C	Pt	I	C
PtI^+	0.47/8.90/0.01	0.50/8.86/0.02	0.61	0.39		0.62	0.38	
$PtI(CH_4)^+$		0.68/8.81/0.02				0.47	0.35	-0.86
TS1		0.68/8.83/0.01				0.47	0.30	-0.72
$PtHI(CH_3)^+$	0.64/8.85/0.02	0.69/8.82/0.01	0.50	0.17	-0.54	0.47	0.31	-0.68
TS2	0.42/9.07/0.02	0.65/8.63/0.02	0.50	0.32	-0.55	0.70	0.24	-0.69
$Pt(CH_3)(HI)^+$	0.44/9.09/0.02	0.64/8.67/0.02	0.46	0.34	-0.56	0.68	0.22	-0.72
TS2-H_2	0.76/8.77/0.03	0.71/8.90/0.02	0.42	-0.01	-0.19	0.37	0.28	-0.44
$PtH_2I(CH_2)^+$	0.76/8.75/0.03	0.71/8.86/0.03	0.45	-0.05	-0.18	0.41	0.28	-0.42
TS3-H_2	0.72/8.77/0.03	0.65/8.90/0.02	0.46	-0.06	-0.08	0.42	0.26	-0.35
$PtI(CH_2)(H_2)^+$	0.67/8.82/0.02	0.68/8.82/0.03	0.48	-0.01	-0.06	0.47	0.27	-0.24
$PtI(CH_2)^+$+H_2	0.66/8.76/0.01	0.70/8.74/0.02	0.58	0.10	-0.07	0.53	0.26	-0.19

Table S5. The single point energy calculation of the reaction PtX^{+}(X=F, Cl, Br, I)+CH_4 at the CCSD(T)/aug-cc-pvtz level.

species	X = F		X = Cl		X = Br		X = I	
	singlet	triplet	singlet	triplet	singlet	triplet	singlet	triplet
PtX^{+}	25.42	0	18.41	0	13.28	0	12.07	0
$PtX(CH_4)^{+}$		-46.20		-36.94				-30.22
TS1		-33.54		-20.64		-69.93		-14.06
$PtHX(CH_3)^{+}$	-64.62	-33.24	-50.64	-18.27	-98.30	-69.53	-39.84	-13.85
TS2	-40.93	-9.75	-36.42	-5.00	-84.44	-49.86	-23.57	
$Pt(CH_3)(HX)^{+}$	-73.93	-48.61	-47.41		-93.13		-31.20	-0.98
$Pt(CH_3)^{+}$+HX	-2.27	-30.62	43.96	15.41	7.02	-21.53	78.49	49.94
TS2-H_2	-63.23	-6.65	-38.04	18.55	-82.03		-18.04	9.68
$PtH_2X(CH_2)^{+}$	-63.20	-6.05	-38.15	20.78	-82.27	-43.63	-18.29	9.47
TS3-H_2	-60.90	-3.89	-36.15	12.52	-80.15	-21.95	-16.00	14.40
$PtX(CH_2)(H_2)^{+}$	-61.50	-10.47	-37.58	7.92	-81.83	-44.85	-17.94	9.35
$PtX(CH_2)^{+}$+H_2	-21.54	16.80	-8.60	14.97	-56.21	-34.40	2.43	20.89

Table S6. Imaginary frequencies (cm^{-1}) of the transition states of the reaction PtX^+(X=F, Cl, Br, I)+CH_4 at the B3LYP/SDD level

species	X = F		X = Cl		X = Br		X = I	
	singlet	triplet	singlet	triplet	singlet	triplet	singlet	triplet
TS1		566.75*i*		516.73*i*		528.18*i*		616.98*i*
TS2	1283.10*i*	924.64*i*	778.46*i*	911.27*i*	663.38*i*	868.53*i*	729.94*i*	625.52*i*
TS2-H2	560.13*i*	513.08*i*	534.43*i*	411.57*i*	564.27*i*	505.79*i*	543.04*i*	595.82*i*
TS3-H2	623.39*i*	864.90*i*	774.35*i*	878.11*i*	808.99*i*	853.06*i*	825.36*i*	792.65*i*

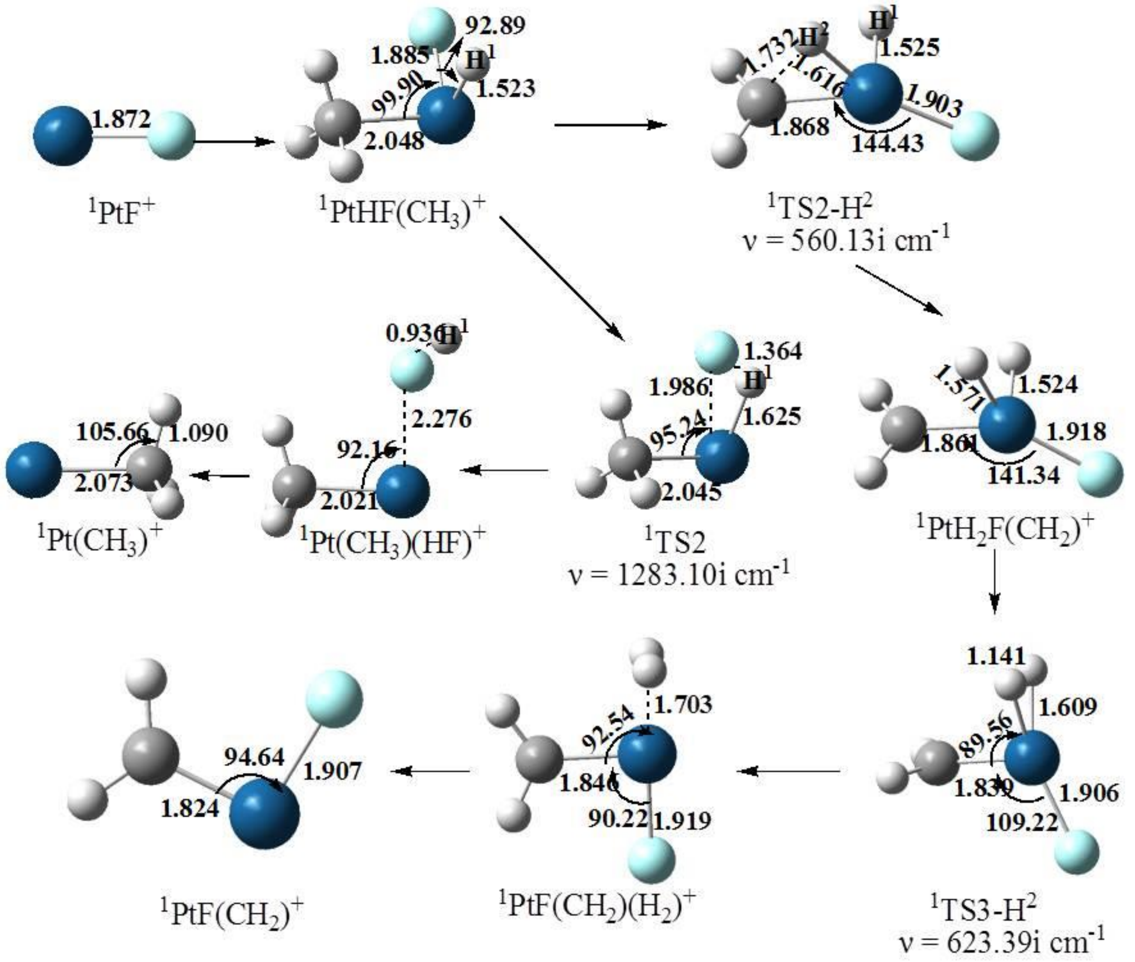

Figure S1. Optimized geometries for the stationary points of the reaction $PtF^+ + CH_4$ in the singlet state (bond lengths in angstroms and bond angles in degrees).

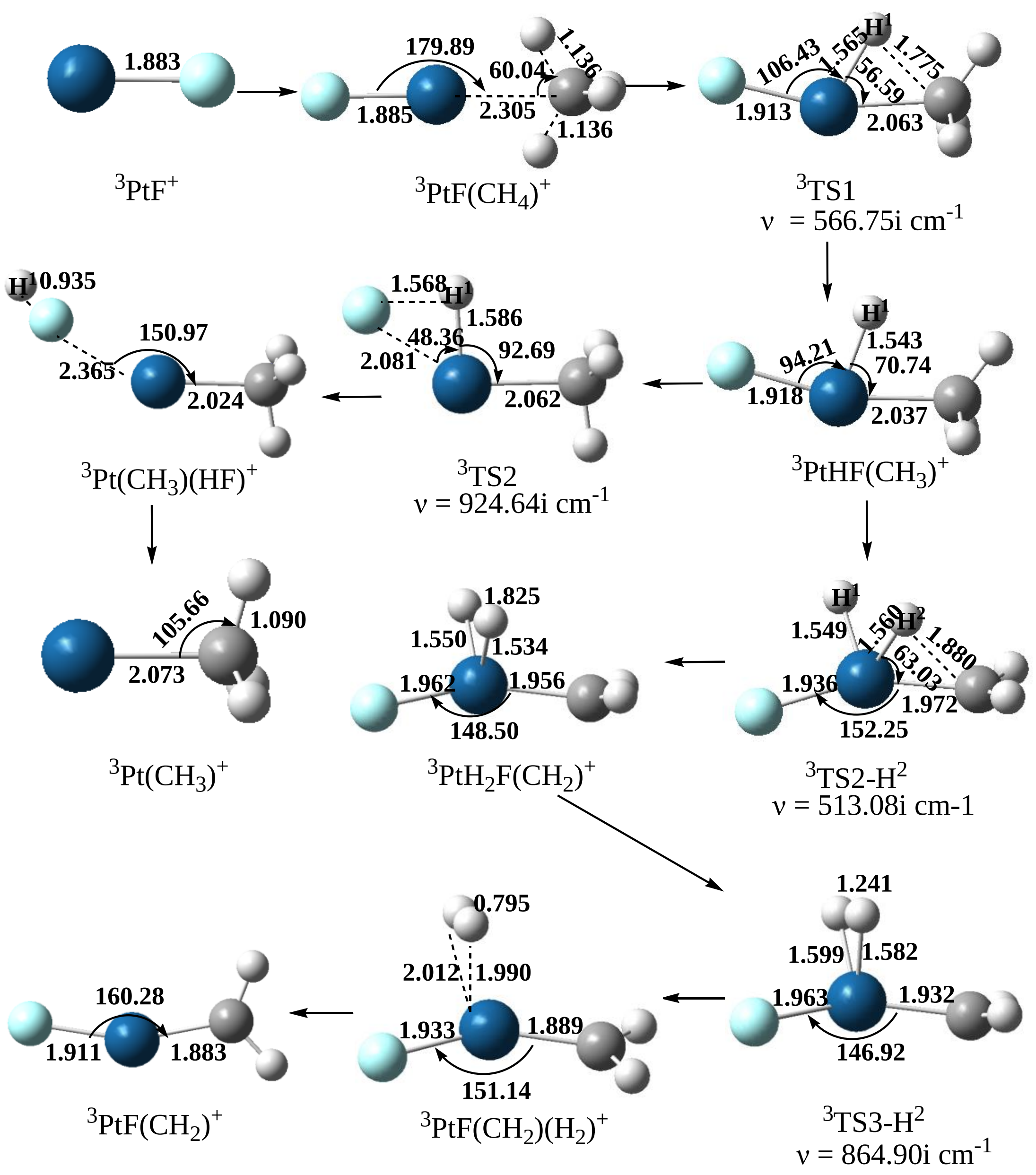

Figure S2. Optimized geometries for the stationary points of the reaction PtF^+ + CH_4 in the triplet state (bond lengths in angstroms and bond angles in degrees).

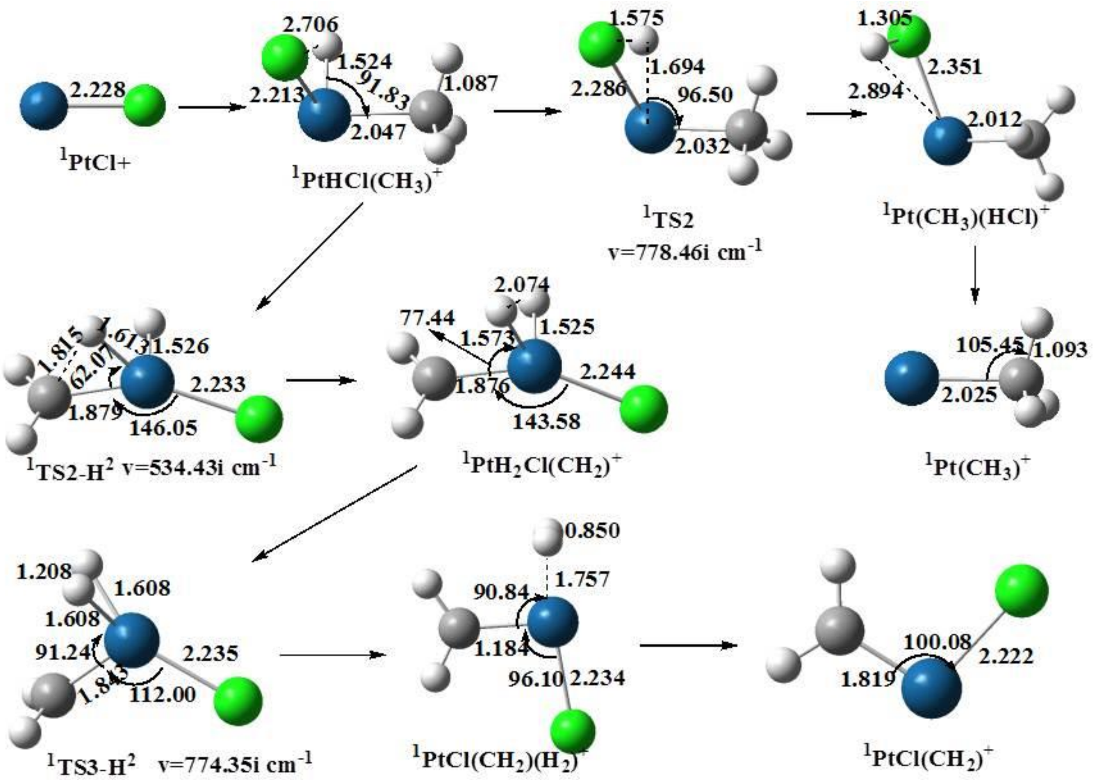

Figure S3. Optimized geometries for the stationary points of the reaction $PtCl^{+}$ + CH_4 in the singlet state (bond lengths in angstroms and bond angles in degrees).

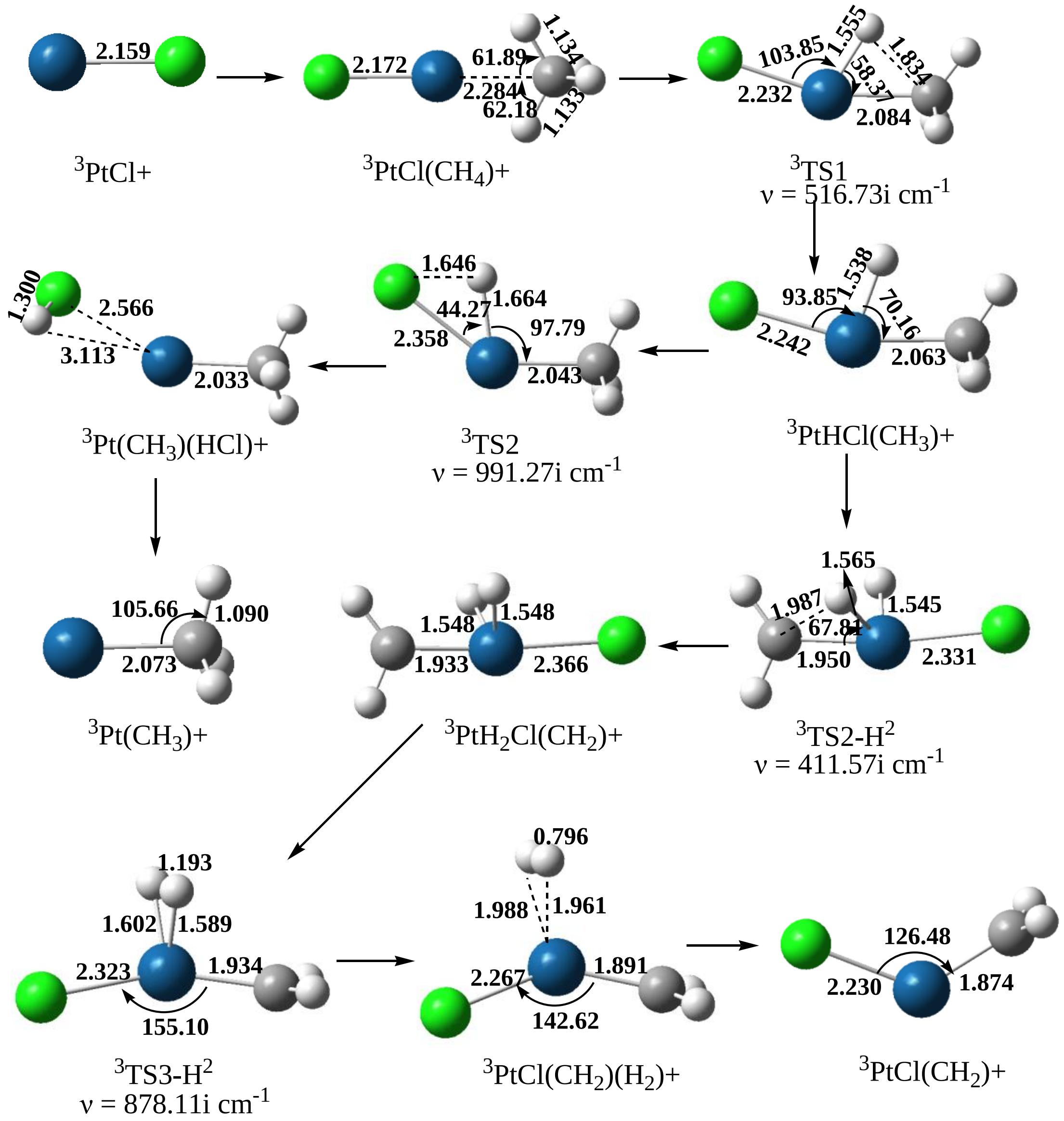

Figure S4. Optimized geometries for the stationary points of the reaction $PtCl^+$ + CH_4 in the triplet state (bond lengths in angstroms and bond angles in degrees).

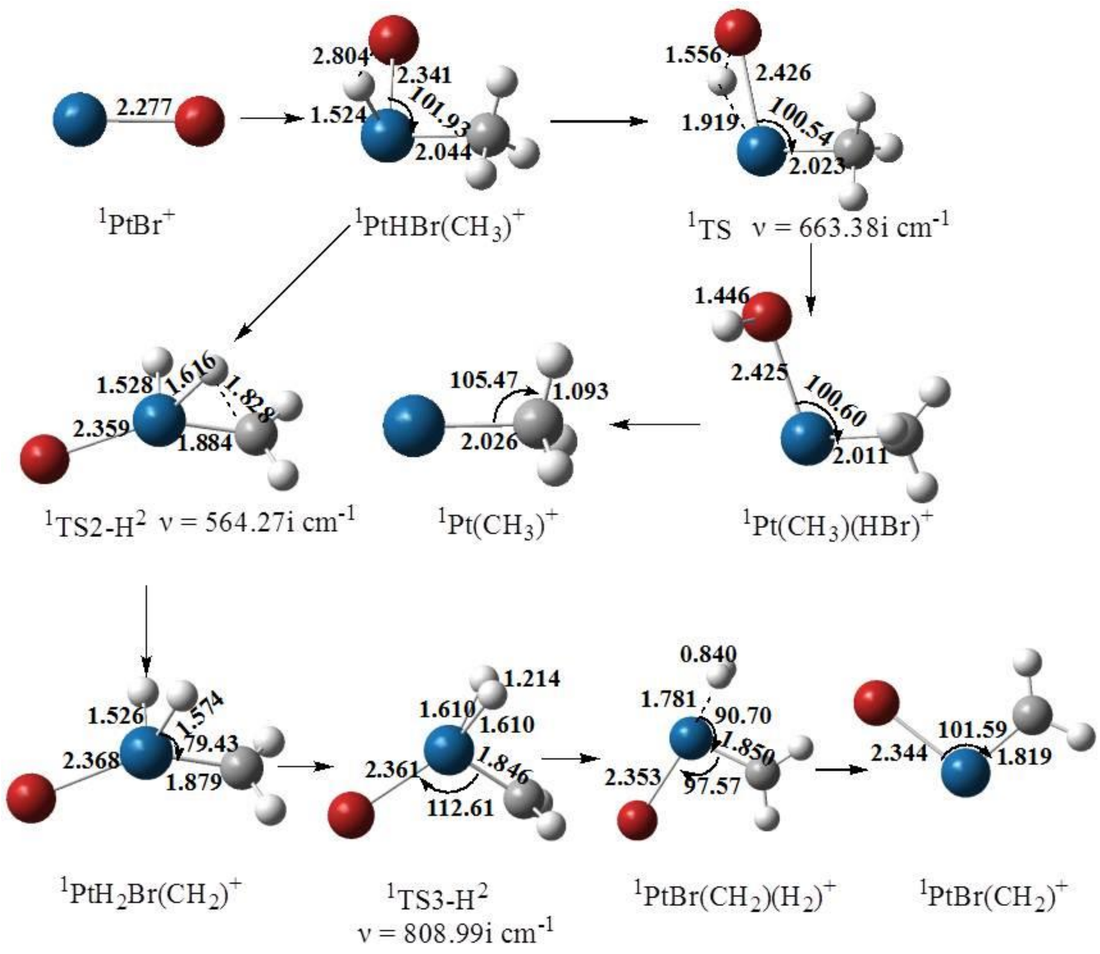

Figure S5. Optimized geometries for the stationary points of the reaction $PtBr^+$ + CH_4 in the singlet state (bond lengths in angstroms and bond angles in degrees).

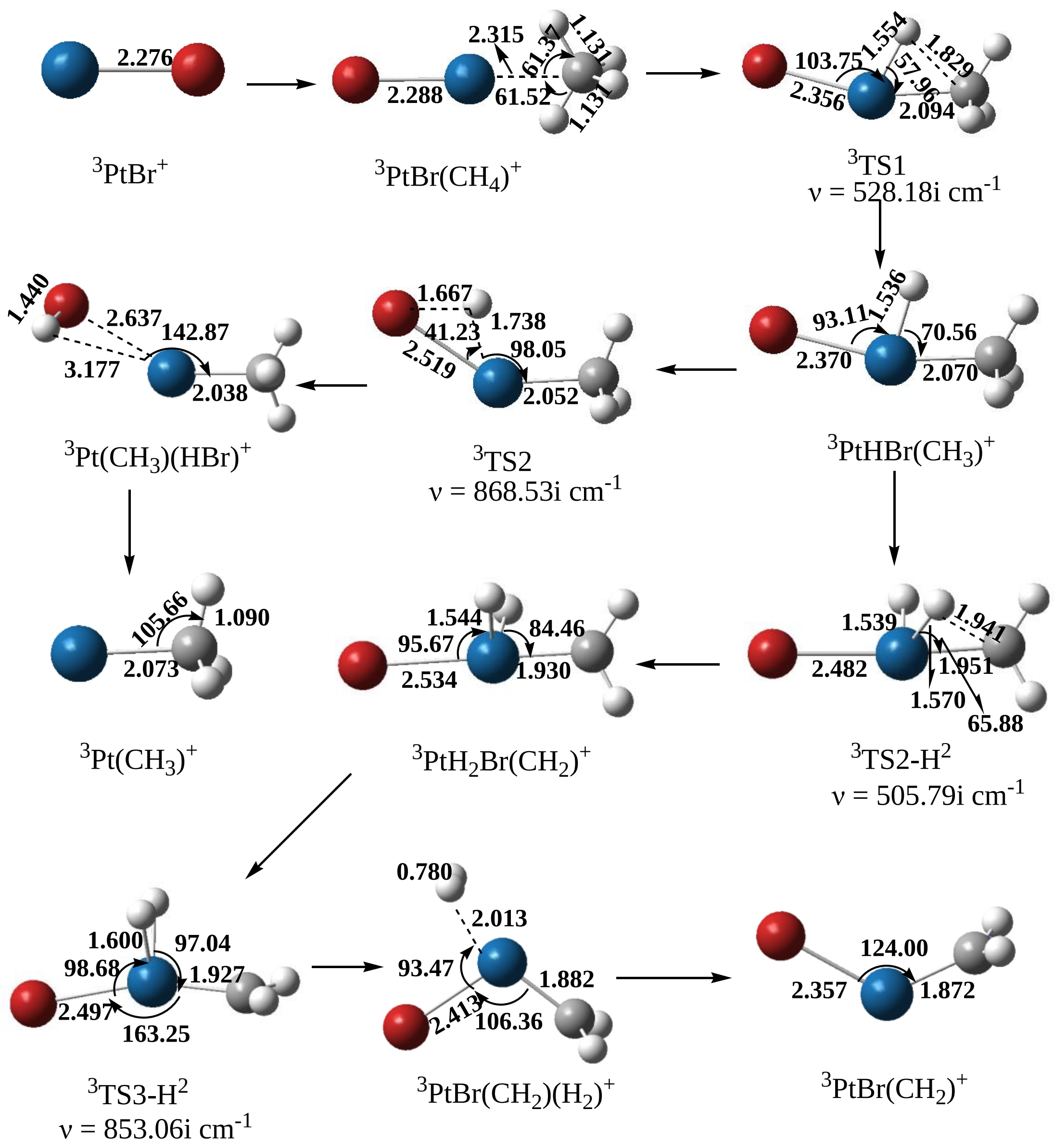

Figure S6. Optimized geometries for the stationary points of the reaction $PtBr^+$ + CH_4 in the triplet state (bond lengths in angstroms and bond angles in degrees).

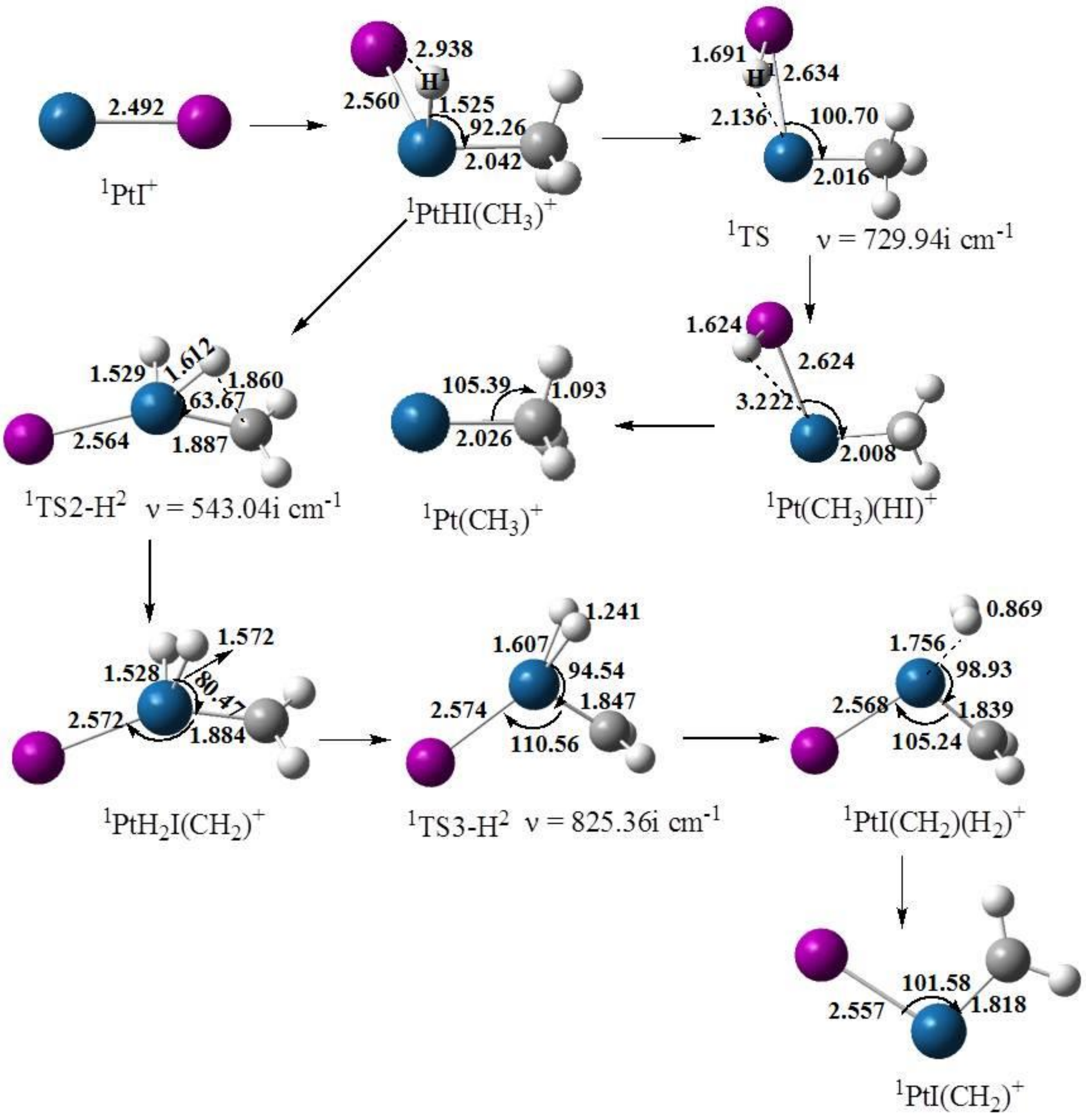

Figure S7. Optimized geometries for the stationary points of the reaction PtI$^+$ + CH_4 in the singlet state (bond lengths in angstroms and bond angles in degrees).

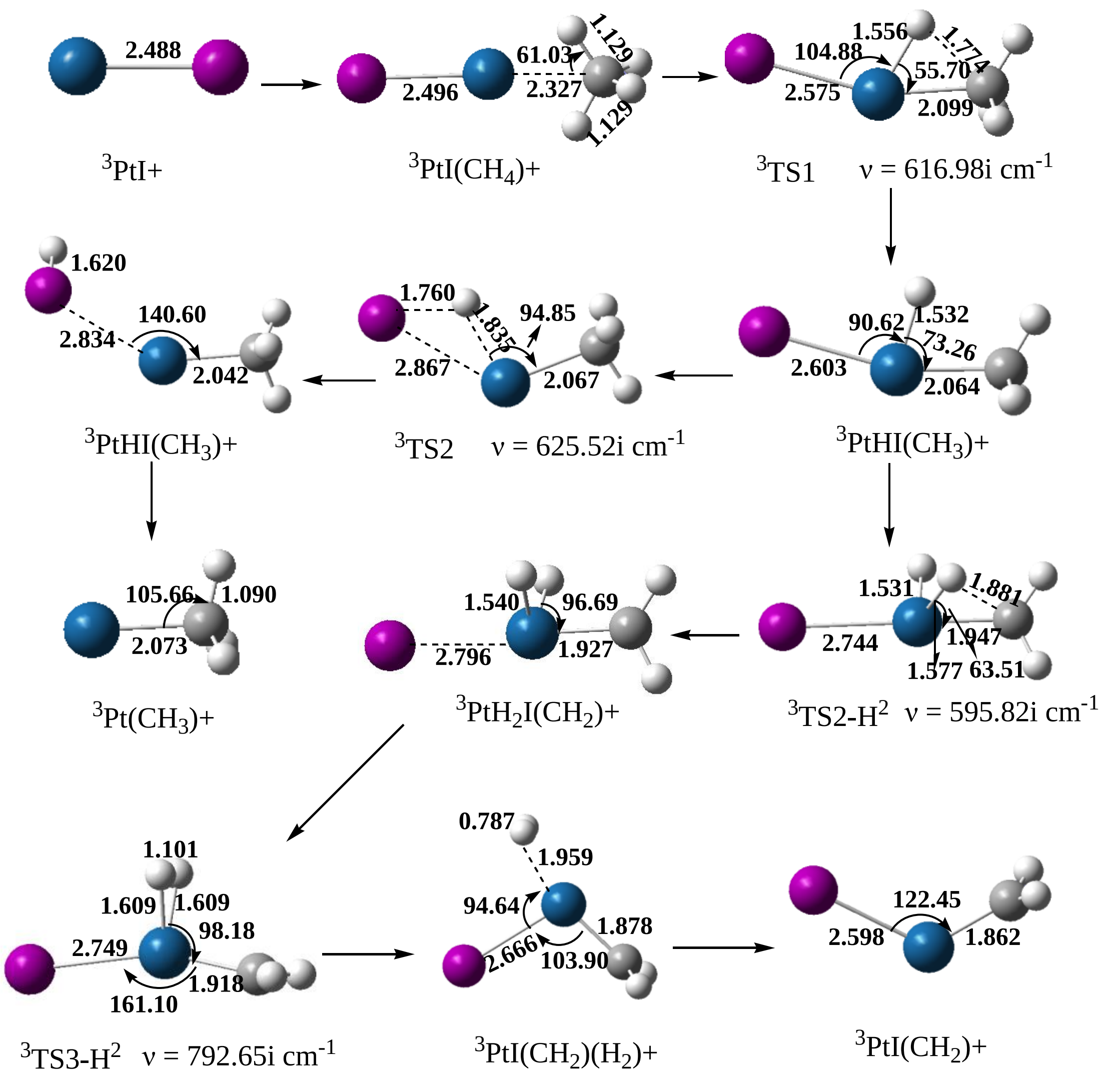

Figure S8. Optimized geometries for the stationary points of the reaction PtI^+ + CH_4 in the triplet state (bond lengths in angstroms and bond angles in degrees).
